# Upcycled Coffee Waste as Sustainable Sorbents for Monitoring Organophosphorus Pesticides in Environmental Waters

**DOI:** 10.1021/acsomega.5c10460

**Published:** 2025-12-22

**Authors:** Saulo Alves de Souza, Gabriel Oliveira Araújo Costa, Grazielle Cabral de Lima, Cristiane Dos Reis Feliciano, Rudy Bonfilio, Mariane Gonçalves Santos

**Affiliations:** † Instrumental Analytical Chemistry Research Group—GPQAI, Institute of Chemistry, 74347 Federal University of Alfenas—Unifal-MG, Alfenas, Minas Gerais 37130-001, Brazil; ‡ Faculty of Pharmaceutical Sciences, Federal University of Alfenas—UNIFAL-MG, Alfenas, Minas Gerais 37130-001, Brazil

## Abstract

This study presents a solid-phase extraction (SPE) method using upcycled coffee waste, specifically acid-treated spent coffee grounds, as sustainable biosorbents for the LC–MS/MS determination of malathion, disulfoton, and chlorpyrifos in environmental waters. Eight coffee-derived materials produced through distinct chemical and thermal pretreatments were fully characterized by FTIR, SEM, TGA, BET, and zeta potential analyses. Acid-treated spent coffee grounds were identified as the most efficient sorbent and subsequently optimized using a 2^5–1^ fractional factorial design. The optimized conditions (conditioning pH 3.0, sample pH 3.0, 25 mg of sorbent, 10 mL of sample, and 0.5 mL of eluent) provided wide linear ranges (5.0–250.0 μg L^–1^ for malathion; 25.0–250.0 μg L^–1^ for disulfoton and chlorpyrifos), low LOQs (5.0 μg L^–1^ for malathion; 25.0 μg L^–1^ for disulfoton and chlorpyrifos), recoveries between 85.9 and 109.3%, and precision with CV ≤15%. The method achieved an AGREEPREP score of 0.64, indicating strong alignment with Green Analytical Chemistry principles. Application to real surface and groundwater samples demonstrated its suitability for detecting organophosphorus residues, confirming the analytical performance and practical applicability of coffee-waste-based sorbents for environmental monitoring.

## Graphical abstract

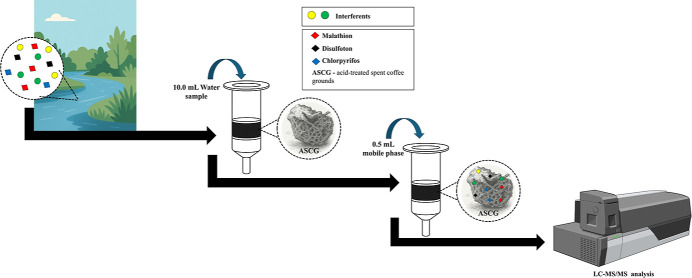

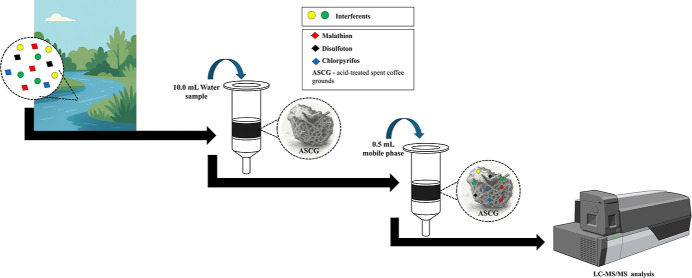

## 1 Introduction

Water is an essential resource for human life and development, but its quality has been compromised by growing pollution resulting from industrialization and urbanization.^1–3^ The main water contaminants include organic and inorganic pollutants, especially toxic metals and organic compounds such as dyes, pharmaceuticals, pesticides, and phenols, which pose risks to human health and the environment.^4,5^ Pesticides have wide agricultural applications and, due to their low selectivity, can cause significant adverse effects on aquatic ecosystems. Organophosphates such as malathion, disulfoton, and chlorpyrifos are widely used and are associated with human poisoning, including fatalities.^6^

The determination of pesticides in environmental samples requires an effective sample preparation step, as these analytes are often found at trace levels in complex matrices that may interfere with detection. Proper pretreatment enhances analyte enrichment, minimizes matrix effects, and increases the selectivity and sensitivity of the method. Therefore, selecting an appropriate sample preparation strategy plays a crucial role in obtaining reliable and reproducible analytical results.^7,8^

In this context, the development of sensitive and sustainable analytical methods for monitoring pesticides in aquatic environments is highly relevant. Among sample preparation strategies, the use of biosorbents has shown promise due to their high adsorption capacity, low cost, and environmental compatibility, consistent with the principles of Green Chemistry. Agricultural residues, particularly from coffee production, have been investigated as biosorbent materials due to their abundance and strong adsorption potential. However, previous studies on coffee-derived biosorbents have mainly focused on the removal of dyes^9,10^ and the extraction of potentially toxic metals^4,5,10,11^ with limited applications to organophosphate pesticides.^12^ Furthermore, unmodified coffee grounds generally exhibit moderate surface area and suboptimal porosity, limiting their adsorption efficiency for organic compounds.^13,14^ Moreover, evaluations of complex environmental matrices and comparisons with conventional extraction methods remain scarce in the literature. This study addresses these gaps by developing a biosorbent based on chemically modified coffee grounds and coffee husks, providing a sustainable and economical alternative to conventional sample preparation methods while maintaining high selectivity and sensitivity for the determination of organophosphorus pesticides.

Several sample preparation techniques have been employed for pesticide determination, each with distinct advantages and limitations. Solid-phase extraction (SPE) offers high selectivity and versatility but can involve relatively complex and time-consuming protocols.^15,16^ Solid-phase microextraction (SPME) minimizes solvent use and sample handling but may be limited by extraction capacity and reproducibility.^17,18^ Liquid–liquid extraction (LLE) is a classic and efficient approach but generally requires large volumes of organic solvents, raising environmental and safety concerns.^19,20^ Dispersive liquid–liquid microextraction (DLLME) provides fast extraction kinetics and low solvent consumption, although solvent selection is crucial for optimal performance.^21^ Molecularly imprinted polymers (MIPs) exhibit high selectivity for target analytes, albeit at the cost of more complex synthesis processes.^22,23^ Restricted access materials (RAMs) facilitate the removal of interfering components from the matrix, but they can be expensive and less available.^24^ Carbon nanotubes (CNTs) offer a large surface area for adsorption and improve extraction efficiency, but their production cost and limited recyclability can pose practical challenges.^25,26^ While these approaches effectively improve analytical performance, their disadvantages include operational complexity, high cost, and, in some cases, environmental impact due to solvent use.

These preparation steps are generally coupled with advanced analytical techniques, such as gas chromatography (GC)^27^ or liquid chromatography (LC),^28^ often combined with tandem mass spectrometry (MS/MS),^29^ providing high sensitivity and selectivity for trace-level detection.^30^

An important factor to consider when discussing pesticides is the significant difference between the permitted limits for residues in drinking water in Brazil and those in the European Union (EU). While the EU takes a precautionary approach, setting a maximum limit of 0.5 μg/L for each pesticide, Brazil allows much higher concentrations, such as 60.0 μg/L for malathion and 30.0 μg/L for chlorpyrifos and disulfoton. This discrepancy is concerning, especially considering that these compounds are acetylcholinesterase inhibitors with proven neurotoxic effects, including cognitive impairment, neurological disorders, and increased risk of chronic diseases.^31,32^

Therefore, this study developed and validated a method for the simultaneous determination of organophosphorus pesticides (malathion, chlorpyrifos, and disulfoton) in environmental waters (groundwater and surface water) using biosorbents derived from coffee production waste, with analysis by liquid chromatography tandem mass spectrometry (LC–MS/MS).^33^

## 2 Materials and Methods

### 2.1 Reagents and Solutions

All reagents used in this study were of analytical or HPLC grade. Citric acid (99%–102%) was obtained from GP Cientfica (Belo Horizonte, Brazil), and sodium hydroxide (NaOH, 99%) was from Synth (Diadema, Brazil). Standard solutions of malathion, disulfoton, chlorpyrifos, and caffeine (≥99%) were purchased from Sigma-Aldrich (Steinheim, Germany). Methanol of HPLC grade (≥99%) was also supplied by Sigma-Aldrich (Steinheim, Germany), and formic acid (85%) was acquired from Biotec (Londrina, Brazil). High-purity deionized water (resistivity of 18.2 MΩ cm) was obtained using a Milli-Q water purification system (Millipore RiOs-DI, Bedford, MA, USA).

### 2.2 Biosorbent Preparation

Coffee waste can be modified by different types of pretreatments to enhance its properties as a biosorbent. In this study, three types of pretreatments were performed: acid, basic, and physical, each with a specific objective. The main objective of basic (alkaline) treatment is to break the bonds between lignin and hemicellulose, exposing functional groups such as hydroxyls and carboxyls, which increase the adsorption capacity and biodegradability of the material. Acid treatment aims to hydrolyze hemicellulose and remove minerals, thereby promoting greater porosity and facilitating the release of sugars, which makes waste more reactive and efficient in adsorption or fermentation processes. Physical treatment promotes the partial degradation of biomass, altering its physical and chemical structures, which results in an increased surface area and enhanced thermal stability, thereby favoring the efficiency of the biosorbent in environmental and industrial applications. Thus, each type of modification contributes in a specific way to improving the characteristics of coffee residue, expanding its potential for use in different processes.^34^

Coffee husk (CH) was sourced from the Machado campus of the Federal Institute of Southern Minas Gerais (IFSULDEMINAS), and SCG was collected by staff following coffee preparation. Initially, both materials were washed with distilled water at 90 °C to remove residual compounds (repeatedly washed until the rinsewater became colorless) and then dried in an oven at 80 °C for 48 h. After being dried, the samples were divided into fractions and subjected to various treatments according to the experimental protocols.

#### 2.2.1 First Process: Carbonization

Portions of 100.00 g of washed and dried spent coffee ground (SCG) and 50.00 g of CH were subjected to carbonization in a muffle furnace at 500 °C under controlled atmospheric conditions to promote thermal decomposition while minimizing oxidative combustion. The materials were maintained at 500 °C for 12 h, resulting in the conversion of organic residues into carbon-rich adsorbents. Following carbonization, the materials were sieved to obtain a uniform particle size distribution and subsequently separated for characterization and application in adsorption studies.

#### 2.2.2 Second Process: Treatment with NaOH

Initially, 50.00 g of washed and dried (SCG) and CH was immersed, separately, in 200.00 mL of 1.0 mol L^–1^ NaOH solution and subjected to magnetic stirring at room temperature (25 °C) for 2 h to facilitate chemical modification. The treated materials were then washed by vacuum filtration with distilled water until a neutral pH was reached, ensuring the removal of residual alkaline compounds. Subsequently, the samples were dried in an oven at 80 °C for 24 h to stabilize the modified adsorbents. This alkaline treatment was intended to introduce hydroxyl (−OH) functional groups onto the adsorbent surfaces, thereby enhancing their adsorption capacity for the target analytes.

#### 2.2.3 Third Process: Treatment with Citric Acid

Initially, 30.00 g of washed and dried SCG and CH samples, individually, was immersed in 200.00 mL of 0.6 mol L^–1^ citric acid solution under continuous magnetic stirring. The suspensions were heated at 60 °C for 12 h to promote interaction between the biomass and citric acid, followed by an increase in temperature to 100 °C to evaporate the solvent until nearly dry. The resulting biosorbents were then extensively washed with distilled water by vacuum filtration to remove noncovalently bound citric acid residues, continuing until the filtrate reached a neutral pH. Finally, the materials were dried at 80 °C for 24 h to stabilize the modified biosorbents. This treatment was designed to functionalize the biosorbent surfaces with carboxylic groups (−COOH), thereby enhancing their adsorption capacity by increasing the number of ion exchange sites.

### 2.3 Characterization of the Adsorbent

Infrared analyses were performed by using a Fourier transform infrared (FTIR) spectrometer (Affinity-1S, Shimadzu) with a spectral resolution of 4 cm^–1^. Spectra were acquired ranging from 4000 to 400 cm^–1^ over 32 scans. The KBr pellet method was employed to enhance spectral resolution, utilizing approximately 1% (w/w) sorbent in the pellet.

Thermogravimetric analyses (TGAs) were conducted on a TG-DTA-SDT Q600 thermogravimetric analyzer (TA Instruments, Castle, USA) from 25 to 800 °C at a heating rate of 10 °C min^–1^ under a nitrogen flow of 100 mL min^–1^.

Scanning electron microscopy (SEM) was conducted at the Federal University of São Carlos (UFSCar) using a Philips XL-30 microscope with a field emission gun (MEV-FEG). The instrument was operated at 10–25 keV, spot size 4, and a working distance of 5 mm. For sample preparation, a small quantity of powder was dispersed in 2.00 mL of isopropanol and sonicated for 10 min, and a drop of the suspension was deposited onto a 5 × 5 mm silicon substrate affixed with double-sided carbon tape. Samples were dried at 40 °C for 12 h before analysis.

Surface area was determined by the Brunauer–Emmett–Teller (BET) method, and pore volume was measured at a *P*/*P*_0_ of 0.90 using the t-plot method. Approximately 110.00 mg of each sample was degassed at 393 K overnight before analysis.

### 2.4 LC–MS/MS Conditions

Analyses were performed using a liquid chromatograph tandem mass spectrometer (LC–MS/MS 8030, Shimadzu, Kyoto, Japan) equipped with a triple quadrupole analyzer and an electrospray ionization (ESI) source. MS/MS parameters were optimized by the direct infusion of 1.0 μg L^–1^ standard solutions of malathion, disulfoton, and chlorpyrifos. The mobile phase consisted of methanol containing 0.1% formic acid and water (80:20, v/v), following established protocols for pesticide quantification by LC–MS/MS, and was delivered at a flow rate of 0.4 mL min^–1^. A 20.00 μL injection volume was used, and all analyses aimed at defining the MS/MS conditions were conducted without the use of a chromatographic column.^35,36^

Analyte-specific parameters, including precursor-to-product ion transitions (*m*/*z*), cone voltage, and collision energy, were manually optimized to maximize the signal intensity. Additional operational parameters were set as follows: interface temperature, 250 °C; heating block temperature, 400 °C; nebulizing gas flow rate, 2.0 L min^–1^; and drying gas flow rate, 15.0 L min^–1^.

Following the optimization of the MS/MS conditions, chromatographic analyses were performed using an XBridge C18 column (2.5 μm, 4.6 × 75 mm) at a flow rate of 0.4 mL/min. The mobile phase consisted of 80% solvent A (methanol with 0.1% formic acid) and 20.0% solvent B (Milli-Q water). The column temperature was maintained at 30 °C, and the injection volume was 20.00 μL. Data acquisition and processing were performed using LabSolutions software.

### 2.5 Selection of Sorbent Based on Analyte Recovery Tests

The selection of the optimal sorbent for analyte extraction in SPE was based on the evaluation of eight coffee-derived materials: CH, SCG, carbonized coffee husk (CCH), carbonized spent coffee grounds (CSCGs), acid-treated coffee husk (ACH), acid-treated spent coffee grounds (ASCGs), base-treated coffee husk (BCH), and base-treated spent coffee grounds (BSCGs). For each test, 5.00 mg of biosorbent was weighed and packed into a commercial SPE cartridge. Using a manifold, 5.00 mL of the sample previously spiked with 1 mg L^–1^ of each pesticide (malathion, disulfoton, and chlorpyrifos), without pH adjustment, was loaded onto the cartridge for the study of analyte retention. Subsequently, 0.50 mL of methanol containing 0.1% (v/v) formic acid was percolated through the cartridge. All tests were performed in duplicate, using the same cartridge for each replicate. As a control, blank extractions were conducted by processing water samples without pesticides under identical conditions.

Extracts were analyzed by liquid chromatography-tandem mass spectrometry (LC–MS/MS) under the previously optimized conditions. The multiple response (MR), as defined in eq 1,^32^ was used as the analytical metric to assess the performance of the coffee-derived sorbents. The material exhibiting the highest MR value was selected for subsequent phases of the study, ensuring optimal extraction efficiency for the target analytes.

1 $$\frac{x}{larger\;area}+\frac{y}{larger\;area}+\frac{z}{larger\;area}=R$$

where *x*, *y*, and *z* represent the peak areas of the analytes of interest (malathion, disulfoton, and chlorpyrifos) obtained for each material, while the denominators correspond to the highest peak area values observed for each respective compound.

### 2.6 pH-Dependent Zeta Potential Analysis

Zeta potential measurements were performed using a Zetasizer Nano ZS equipped with an MPT-2 Titrator (Malvern, Worcestershire, UK) and conducted exclusively for the material exhibiting the highest extraction performance. Aqueous suspensions (10.00 mL) containing 5.0 mg mL^–1^ of ASCG were sonicated for 30 min to ensure proper dispersion. Subsequently, 0.2 mL of the suspension was diluted in 10.00 mL of 0.020 mol L^–1^ phosphate buffer at varying pH values (3.0–11.0) prior to analysis.

### 2.7 Adsorption Kinetics and Isotherm

To perform the adsorption kinetics, solutions of malathion, chlorpyrifos, and disulfoton were prepared in methanol separately at a concentration of 100.00 μg L^–1^. The following experimental time points were used: 0, 0.25, 0.50, 0.75, 1.00, 5.00, 10.00, 20.00, and 30.00 min. The experiments were performed in triplicate, and a blank sample containing water was also analyzed. For the procedure, 1.50 mL of each pesticide standard solution was placed separately in contact with 25.00 mg of ASCG in glass tubes, which were kept under agitation at 1000 rpm for the specified periods. After the corresponding times, the tubes were centrifuged at 2000 rpm, and the supernatants were subsequently collected, filtered, and analyzed by LC–MS/MS to determine the concentrations of malathion, chlorpyrifos, and disulfoton in the remaining solutions. The mass of analyte adsorbed by the material was calculated considering the difference between the initial total mass of analyte before adsorption and the mass remaining in the supernatants, relative to the total mass of ASCG used, according to eq 2

2 $$q=\frac{\left(C_{0}-C_{f}\right)\cdot V}{m}$$

where *C*_0_ (mg L^–1^) is the initial concentration, *C*_e_ (mg L^–1^) is the analyzed concentration, *V* (L) is the volume of the malathion, chlorpyrifos, and disulfoton solutions, and *m* (g) is the mass of the sorbent.^37^

The data were processed according to pseudo-first-order, pseudo-second-order, chemisorption (Elovich), and fractional-order models and fitted using a nonlinear fitting method with 64 bit OriginPro 2018 software (OriginLab, United Kingdom), considering the linear correlation coefficient (*R* ^2^) values. To confirm the best fit, the models were statistically evaluated based on the error function (*F*_error_) (eq 2), which correlates the theoretical amount of malathion, chlorpyrifos, and disulfoton adsorbed by the material with the experimental measurements, considering the number of parameters in the fitted model^37^

3 $$F\;error=\sqrt{\left(\frac{1}{n-p}\right)\sum_{i}^{n}{\left(q_{i},\exp-q_{i},theoretical\right)}^{2}}$$

where *n* is the number of experiments performed, *p* is the number of parameters of the fitted model, *q*_*i*_,exp is each value of q measured experimentally and *q*_*i*_,theoretical is each value of q predicted by the fitted model.

The adsorption isotherms were constructed using solutions of malathion, chlorpyrifos, and disulfoton prepared in methanol, separately. The following concentrations were evaluated: 100.00, 250.00, 500.00, 1000.00, 3000.00, 5000.00, 8000.00, 10000.00, and 20000.00 μg L^–1^ (in triplicate for each concentration). 1.5 mL portion of each pesticide standard solution was contacted with 25.00 mg of ASCG in test tubes and shaken at 1000 rpm for 1 min. After that, the tubes were centrifuged at 2000 rpm, the supernatants were subsequently collected, filtered, and analyzed by LC–MS/MS. The mass adsorbed by the material was calculated considering the difference between the initial total mass of analyte before adsorption and the mass remaining in the supernatants, relative to the total mass of ASCG used (eq 2). All data were fitted according to the Langmuir, Freundlich, Sips, Khan, Tóth, and Redlich–Peterson isotherm models using nonlinear fitting with OriginPro 2019 64 bit software (OriginLab, UK), based on the linear correlation coefficient (*R* ^2^) values and *F*_error_ (eq 3).

### 2.8 Optimization of Extraction Conditions

Environmental water samples (groundwater and surface water) free of the analytes were collected from the Alfenas (Minas Gerais, Brazil) region, carefully filtered to remove particulates, and subsequently pooled by combining five individual samples for analysis. The pooled sample was spiked with a mixture containing the three target pesticides at 1.00 mg L^–1^. For optimization, varying masses of ASCG biosorbent were individually weighed and packed into a commercial SPE cartridge. The cartridge was conditioned by percolating 1.00 mL of pH-adjusted ultrapure water by using a manifold. Following conditioning, different volumes of the pooled sample (adjusted to different pH values) were loaded onto the BCH-packed cartridge at a flow rate of 1 mL min^–1^. The analytes were eluted with different volumes of methanol with 0.1% formic acid, and the eluate was filtered through a 0.45 μm syringe filter before analysis by LC–MS/MS under previously optimized conditions.

Blank analyses using Milli-Q water were also performed in triplicate to assess potential interferences from the sorbent material.

Optimization of the extraction conditions was performed using a 2^5–1^ fractional factorial design, evaluating the following independent variables: conditioning pH, sample pH, sorbent mass, sample volume, and eluent volume. The multiple response (MR) served as the dependent variable. A total of 19 experiments were conducted, including triplicates at the central point to estimate experimental error (Table S10, in the Supporting Information). Data analysis was performed using the StatSoft STATISTICA 10.0 software package (Tulsa, Tulsa, USA).

The optimized conditions for the developed method were as follows: sample pH adjusted to 3.0; conditioning pH, 3.0; biosorbent mass, 25.00 mg; elution volume, 0.50 mL of methanol containing 0.1% formic acid; and sample volume, 10.00 mL.

### 2.9 Evaluation of the Cleanup in the Extraction Process

To enhance detection sensitivity and minimize potential interferences, a washing step was incorporated after sample loading and before elution. The extraction procedure involved sequential percolation of 1.00 mL of Milli-Q water, 5.00 mL of the pooled sample fortified with pesticides, an additional 1.00 mL of Milli-Q water for washing, and finally 0.50 mL of eluent (methanol with 0.1% formic acid). The resulting extracts were analyzed by LC–MS/MS. The necessity of the washing step was evaluated by determining whether the peak areas of the pesticides and caffeine were significantly reduced. A reduction in the caffeine signal, accompanied by consistent analyte signal intensity, would suggest that the cleanup step was efficient in decreasing the level of interferents and mitigating ion suppression.

### 2.10 Evaluation of Memory Effect

To assess the memory effect, a cartridge washing step was evaluated. Ten consecutive extractions were performed, and after each extraction, 1.00 mL of methanol acidified with 0.1% formic acid was percolated through the cartridge before subsequent extraction. The extraction procedure followed the protocol described in Section 2.8. The resulting extracts were analyzed by LC–MS/MS.

### 2.11 Evaluation of Matrix Effect

The presence of matrix effects in the method was evaluated by constructing calibration curves for malathion, chlorpyrifos, and disulfoton using both standard solutions in solvent and matrix-matched extracts. Qualitatively, the matrix effect was assessed by comparing the linear regression lines of the respective calibration curves. Quantitatively, the matrix effect was determined according to eq 4.

4 $$ME\;\left(\%\right)=\frac{S_{extract}-S_{solvent}}{S_{solvent}}\times 100$$

where *S*_solvent_ refers to the slope of the calibration curve constructed from pesticide standards prepared in solvent, and *S*_extract_ refers to the slope of the calibration curve constructed from pesticide standards prepared in the matrix extract.

### 2.12 Figures of Merit

The analytical performance of the developed method was evaluated according to key parameters, including linearity, limit of detection (LOD), limit of quantification (LOQ), precision, and accuracy. These parameters were assessed under optimized conditions to ensure the reliability and applicability of the SPE-LC-MS/MS method for the determination of pesticides in environmental water samples. The analyses were conducted according to the European Union guideline SANTE/11312/2021.^38^

Linearity was evaluated by constructing matrix-matched calibration curves using six concentration levels: 5.0, 10.0, 50.0, 100.0, 150.0, and 250.0 μg L^–1^ for malathion and 25.0, 50.0, 75.0, 100.0, 150.0, and 250.0 μg L^–1^ for disulfoton and chlorpyrifos. Each concentration was analyzed in six replicates, and calibration curves were obtained by plotting the analyte signal as a function of the concentration.

Accuracy was evaluated based on recovery (%), while precision was assessed using the coefficient of variation (CV). The following concentrations 5.0, 100.0, and 150.0 μg L^–1^ were analyzed for malathion and 25.0, 100.0, and 150.0 μg L^–1^ for disulfoton and chlorpyrifos. The method was deemed accurate and precise when recovery ranged from 80% to 120% and CV values were ≤20% at the LOQ, and recovery remained between 85% and 115% with CV values ≤15% at higher concentrations. These criteria ensure the reliability of the measurements and compliance with acceptable error margins.

The limits of detection (LOD) and quantification (LOQ) were determined experimentally. The LOD is defined as the lowest concentration of the analyte that can be reliably distinguished from the background (or blank), representing the lowest concentration at which the presence of the analyte can be detected but is not necessarily quantified. The LOQ, in contrast, is the lowest concentration at which the analyte can be reliably quantified with an acceptable accuracy (typically 85.0 and 115.0%) and precision (relative standard deviation ≤15.0%).

### 2.13 Application in Real Samples

After optimization and validation, the developed method was applied to 14 environmental water samples collected in the Alfenas region (Minas Gerais, Brazil), including groundwater and surface water from artesian wells and the Furnas reservoir. The samples were collected near agricultural areas suspected of pesticide contamination. After collection, the samples were carefully filtered, transferred to 100 mL amber glass bottles, and stored under refrigeration until analysis. Before extraction, the pH of each sample was adjusted to 3.0 by using 0.1 mol L^–1^ formic acid, and no additional pretreatment was required.

## 3 Results and Discussion

### 3.1 Syntheses and Characterizations of Biosorbents

The materials were initially characterized by Fourier transform infrared spectroscopy (FTIR) to identify the functional groups present in the coffee-derived materials. The FTIR spectra of CH and SCG exhibited characteristic bands at 3348 cm^–1^, corresponding to O–H stretching vibrations; 2916 cm^–1^, attributed to aliphatic C–H stretching; 1735 and 1650 cm^–1^, corresponding to C = O stretching vibrations; 1443 cm^–1^, associated with C–H bending in – OCH_3_ groups; 1257 cm^–1^, assigned to C–O stretching vibrations; and 1051 cm^–1^, corresponding to C–O–C stretching vibrations. These bands are characteristic of cellulose, hemicellulose, and lignin present in the biomass (Figure 1).^39–41^

**Figure 1:**
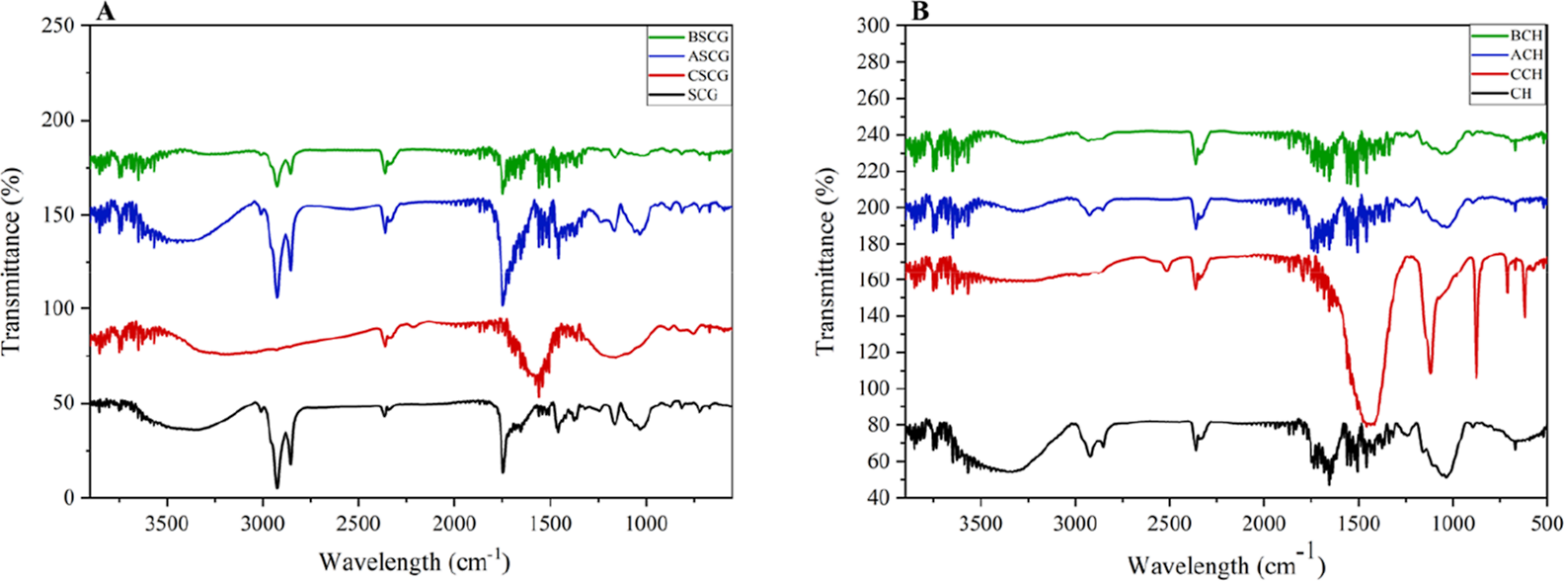
FTIR spectra of (A) coffee grounds and (B) coffee husk after respective treatments.

The FTIR spectra of ACH, ASCG, BCH, and BSCG displayed bands similar to those observed in the CH and SCG spectra; however, a decrease in band intensities was noted. The band at 1051 cm^–1^, attributed to C–O–C stretching of the glycosidic bonds in cellulose and hemicellulose, exhibited a decrease in intensity, indicating possible degradation of these macromolecules due to the hydrolytic action of the treatments. Additionally, the reduction in intensity in the 2900–3500 cm^–1^ region, corresponding to aliphatic C–H (∼2925 cm^–1^) and O–H (∼3348 cm^–1^) stretching vibrations, suggests the cleavage of polymer chains and the removal of hydroxyl and aliphatic groups, potentially accompanied by the elimination of volatile compounds.^42^

Moreover, for ASCG, the FTIR spectra revealed features consistent with the incorporation of carboxylic groups (−COOH). This modification is expected because citric acid promotes esterification and surface functionalization during heating. In these samples, the C=O stretching band in the 1735–1650 cm^–1^ region became more defined relative to the untreated materials, indicating an increased contribution of carbonyl groups associated with protonated carboxylic functionalities.^43^ These changes corroborate the surface chemistry described in the preparation step, where acid treatment introduces −COOH groups capable of enhancing adsorption through hydrogen bonding and nonelectrostatic interactions.

The FTIR spectrum of BSCG specifically exhibited a decrease in intensity of the band at 1750 cm^–1^ (C=O stretching of hemicellulose and lignin), as well as reductions in intensities of the bands at 1257 cm^–1^ (C–O bonds of lignin) and 1040 cm^–1^ (C–O–C bonds of cellulose and hemicellulose), suggesting degradation and removal of soluble fractions of these biopolymers.^44^

In the FTIR spectra of CCH and CSCG, an increase was observed in the intensity of the band at about 1500 cm^–1^, indicating C=C stretching in conjugated structures, formed by chemical restructuring induced by thermal treatment, including the elimination of oxygenated groups and the formation of new molecular structures.^45^ Carbonization also resulted in more pronounced structural changes, such as the disappearance of bands between 2750 and 3000 cm^–1^ (aliphatic C–H), reduction in the intensity of bands at 3348 cm^–1^ (O–H) and 1040 cm^–1^ (C–O–C), and increased intensity in the regions between 2000–1667 cm^–1^ (overtones and combination bands of aromatic rings). These changes indicate the formation of more aromatic and hydrophobic structures.^45^ Such structural transformations directly influence the surface properties of the treated materials, enhancing their efficiency for organic compound adsorption and improving their performance as biosorbents.

The biosorbents were further characterized by thermogravimetric analysis (TGA). Figure 2 presents the TGA and derivative thermogravimetric (DTG) curves for CH, CCH, ACH, and BCH. All materials exhibited three main mass loss events. The first event, observed below 200 °C, is attributed to the removal of moisture and volatile compounds; this process was most pronounced in CCH. The second event, occurring between 200 and 400 °C, corresponds to the thermal decomposition of hemicellulose, cellulose, and a portion of lignin, resulting in significant mass loss. ACH and BCH exhibited only minor behavioral differences compared to CH in the temperature range of 200–400 °C. This observation is consistent with the FTIR results, which showed a reduction in the intensity of bands associated with cellulose, hemicellulose, and lignin; however, these bands remained detectable in the corresponding FTIR spectra. In contrast, CCH did not show significant thermal degradation within this temperature range, supporting the FTIR findings that indicate probable chemical degradation of cellulose, hemicellulose, and lignin because of the carbonization process applied to CCH. The third event, above 400 °C, is characterized by a reduced degradation rate and is attributed to the residual decomposition of lignin. The CCH sample demonstrated greater thermal stability due to prior carbonization, which removed most volatile matter and produced a more stable carbonaceous residue. Above 600 °C, mass loss stabilized, indicating the completion of organic compound degradation. Overall, chemical treatments and carbonization significantly influenced the thermal properties of the samples, enhancing their suitability for energy and adsorption applications due to improved high-temperature stability.^46^

**Figure 2:**
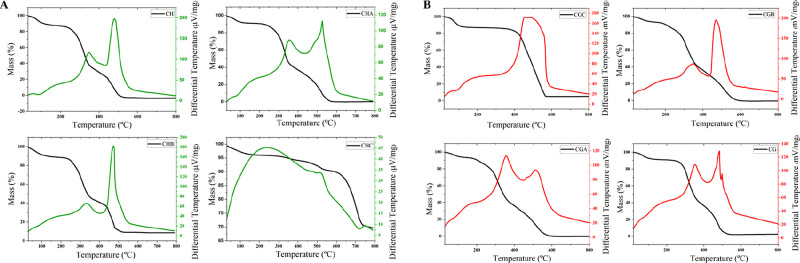
TGA and DTG analyses of (A) coffee husk and (B) coffee grounds.

The TGA and DTG profiles for SCG, CSCG, ASCG, and ASCG (Figure 2) similarly revealed a three-stage mass loss process. The first stage, below 200 °C, corresponds to the removal of moisture and light volatiles and is endothermic.^47^ CSCG did not exhibit any mass loss between 200 and 400 °C, unlike SCG, ASCG, and BSCG, which showed noticeable degradation within this range. This indicates that the carbonization process led to the degradation of cellulose, hemicellulose, and lignin in CSCG, as also supported by the FTIR spectra. As a result, only thermally stable compounds, those that degrade above 400 °C, remained in the carbonized coffee grounds. Compared to the husk samples, the carbonized grounds appear to contain even less organic matter that degrades within the 200–400 °C range, as evidenced by the near absence of mass loss in this region, whereas the husk still exhibited a slight mass loss. In the third stage (above 400 °C), the degradation rate decreases due to the complex structure of lignin, with the remaining mass corresponding to carbonaceous residue; mass loss stabilizes above 600 °C. In summary, the initial dehydration event is endothermic, while subsequent decomposition events are exothermic.^46^

The surface morphology of the treated biosorbents, revealed by scanning electron microscopy (SEM), shows distinct differences between the raw and modified materials. Untreated coffee husk (CH) (Figure 3) and coffee grounds (SCG) (Figure 4) exhibit compact and irregular surfaces with a low porosity. In contrast, the pretreated biosorbents present markedly more porous and heterogeneous structures, highlighting the effectiveness of chemical and thermal treatments in altering the surface morphology and increasing the adsorption potential. The acid-treated husk (ACH) exhibited irregular lamellar layers and heterogeneous pore sizes, probably resulting from alterations in lignocellulosic structures and functional groups induced by the acid treatment, as seen in Figure 3B. The alkali-treated husk (BCH) in Figure 3C showed similar characteristics but with fewer lamellae and greater porosity due to alkaline hydrolysis. The carbonized shell (CCH) exhibited a rudimentary porosity characterized by cavities of varying sizes formed by heat treatment, substantially increasing the surface area and generating additional adsorption sites (Figure 3D). Similarly, the acid-treated grains (ASCG) (Figure 4F) and the alkali-treated grains (BSCG) (Figure 4G) exhibited honeycomb-shaped lamellar networks with heterogeneous pores and reduced lamellae after selective degradation and alkaline hydrolysis, respectively. The carbonized grains (CSCG) developed robust and irregularly distributed cavities that formed an extensive porous network, significantly increasing the surface area and the adsorption potential of organic contaminants, as represented in Figure 4H.

**Figure 3:**
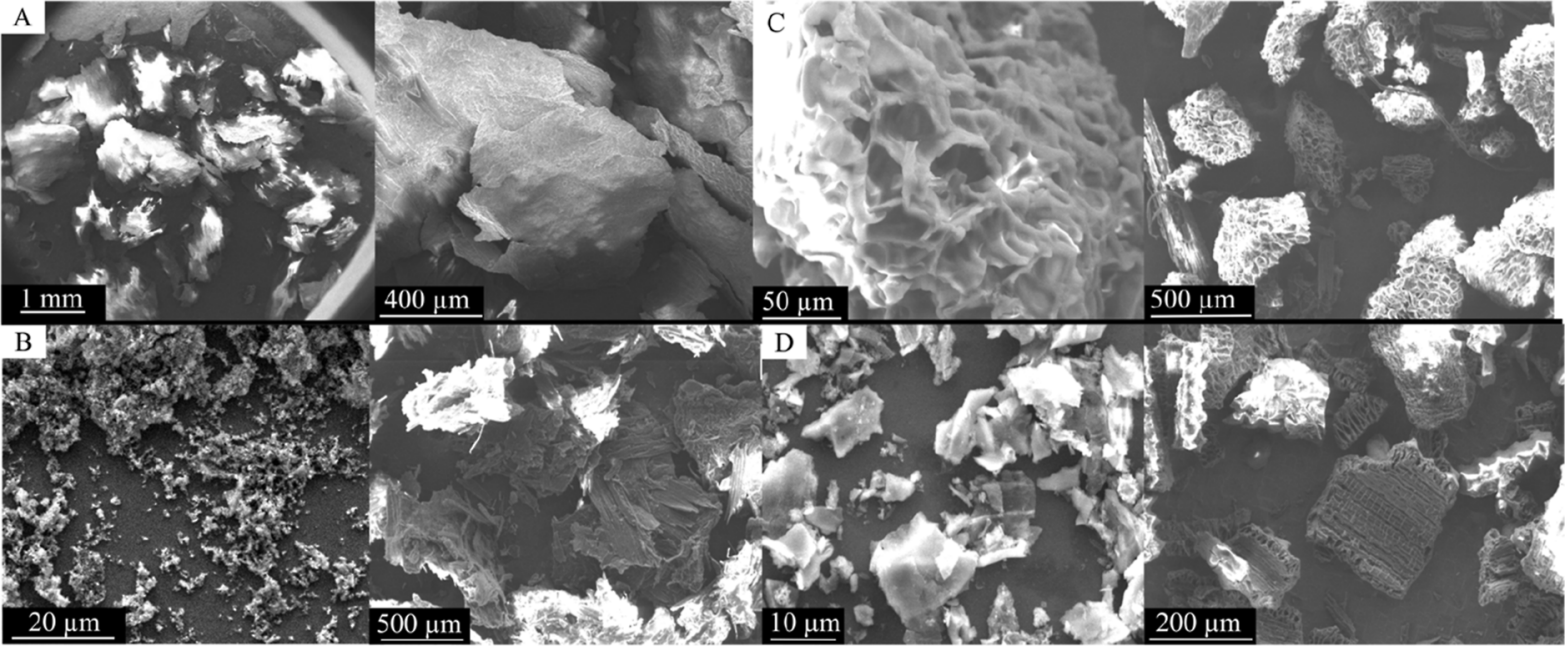
surface morphology of the treated biosorbents by scanning electron microscopy. (A) CH, (B) ACH, (C) BCH, and (D) CCH.

**Figure 4:**
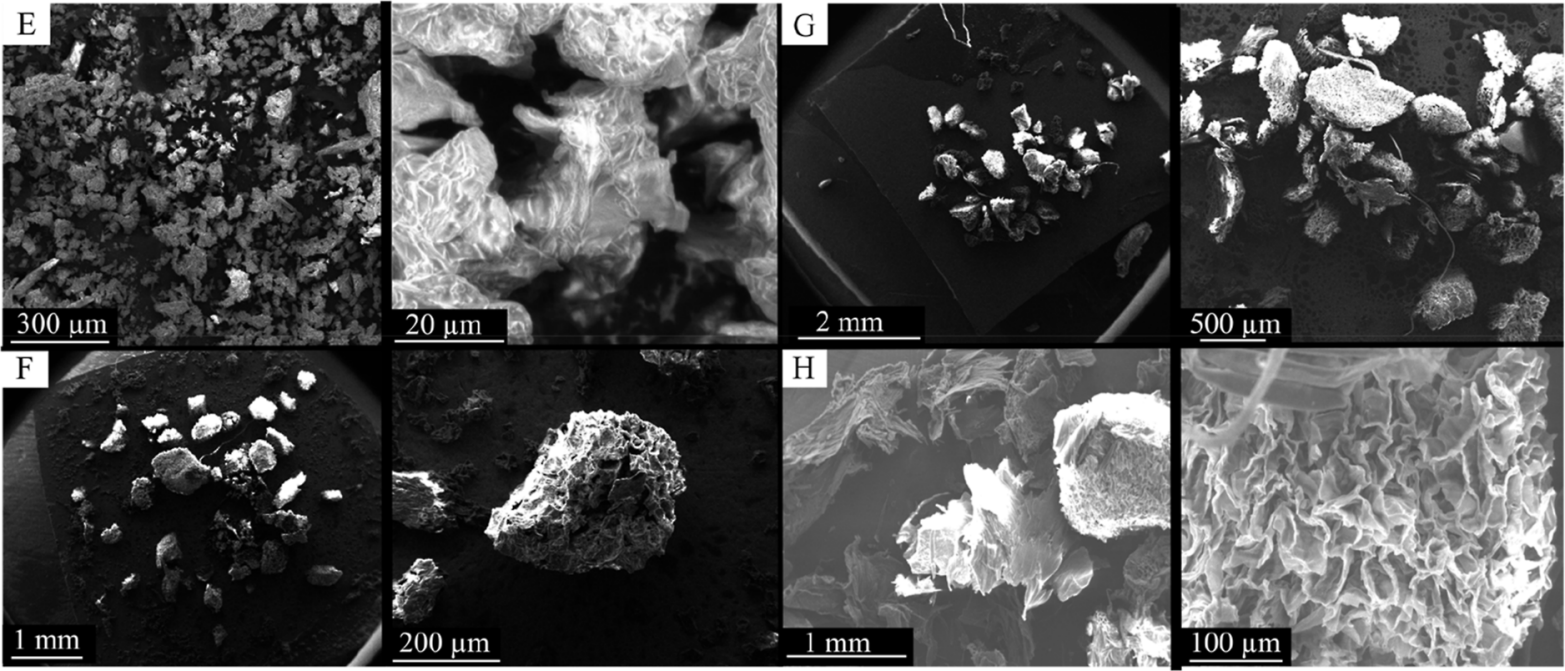
The surface morphology of the treated biosorbents by scanning electron microscopy. (E) SCG, (F) ASCG, (G) BSCG, and (H) CSCG.

The Brunauer–Emmett–Teller (BET) method was used to determine the specific surface area, total pore volume, pore size distribution, and isotherm type of CH, SCG, CCH, CSCG, ACH, ASCG, BCH, and BSCG (Table S1 and Figure S1 in the Supporting Information). The BET results revealed clear differences in the pore structure and adsorption behavior among the materials, as reflected by their isotherm profiles and textural properties. Materials exhibiting type III isotherms (ACH, ASCG, BCH, BSCG, SCG, and CSCG) generally showed low specific surface areas and pore diameters in the microporous to fine-mesoporous range, consistent with weak adsorbate–adsorbent interactions and adsorption governed predominantly by multilayer formation. CSG distinguished itself by displaying a substantially higher surface area and larger pore size relative to those of the other type III materials, suggesting a greater capacity to accommodate larger molecules. CCH, characterized by a type II isotherm and the highest specific surface area among the mesoporous samples, exhibited an unrestricted multilayer adsorption behavior typically associated with nonporous or macroporous materials with accessible external surfaces. In contrast, CH displayed a type IV isotherm with a discernible hysteresis loop, indicative of capillary condensation within a narrow mesoporous structure and confirming its suitability for adsorbing small- to medium-sized molecules.

According to IUPAC classification, materials with pore diameters <2 nm (CH, SCG, ASCG, and BSCG) are classified as microporous and tend to favor selective adsorption of smaller molecules, whereas those with larger pore diameters (BCH, ACH, CCH, and CSG) fall within the mesoporous domain and provide higher adsorption capacity for bulkier compounds.^48^

Comparative analysis of the specific surface area and pore volume showed that CSCG underwent a significant increase following carbonization, suggesting that the thermal treatment enhanced pore development and generated a more open porous network. In contrast, both acid and base treatments resulted in reductions in surface area and pore volume in ACH, ASCG, BCH, and BSCG compared with their untreated precursors (CH and SCG). This reduction may be attributed to the introduction of new surface functional groups during chemical modification, which may partially block pore entrances or occupy adsorption sites, thereby decreasing the apparent porosity.

Although specific surface area, pore volume, and pore diameter are frequently correlated to the adsorption capacity of materials, these factors should not be evaluated in isolation when selecting the most suitable adsorbent for a specific class of chemical compounds. Instead, they should be evaluated in conjunction with other physicochemical properties of the adsorbents. In this context, FTIR and TGA characterizations revealed structural differences between the materials, providing information about their surface chemistry. The presence of functional groups associated with cellulose, hemicellulose, and lignin plays an important role in the interaction and adsorption of analytes, thus facilitating the extraction process and, most importantly, contributing to a more effective adsorbent–adsorbate interaction.

### 3.2 Optimization of LC–MS/MS Conditions

The MS/MS parameters were optimized by direct injection of standard solutions of the analytes, previously prepared in methanol at a concentration of 1.0 μg L^–1^. The specific parameters for each analyte, such as monitoring of selected reaction transitions (SEM), collision energy (CE), and cone voltage (CV), were adjusted to maximize the intensity of the analytical signal, as shown in Table S2 in the Supporting Information. The positive ionization mode was selected for malathion, chlorpyrifos, and disulfoton.

In addition to the parameters mentioned above, several other LC–MS/MS conditions were thoroughly evaluated to ensure optimal chromatographic separation, ionization efficiency, and detection sensitivity. These additional parameters, which contribute to the overall robustness and reliability of the analytical method, are summarized in Table 1.

**Table 1:** Optimization of LC–MS/MS Parameters

LC parameters	description	MS parameters	description
column	XBridge C18 2.5 μm 4,6 × 75 mm	ion source	ESI
temperature	30 °C	ionization mode	+
mobile phase A	MeOH +1% formic acid	capillary voltage	0 kV
mobile phase B	water	source temperature	250 °C
elution mode	isocratic	gas temperature	250 °C
flow rate	0.4 mL min^–1^	nebulizing/drying gas flow	1,5 L min^–1^/12 L min^–1^
injection volume	5 μL	analyzer type	triple quadrupole
total analysis time	12 min	acquisition mode	MRM
type of autosampler	SIL-20A	collision energy	–25 V
		dwell time	100 ms
		collision gas	argon

The identification criteria included the simultaneous observation of both fragments of each molecule, the comparison of the relationship between these fragments with the standard analyses of the pesticides, and the evaluation of the relative abundances of the fragments. The quantitative analyses were carried out using the SRM (selected reaction monitoring) transition with the highest intensity (malathion: transition 331 → 127; disulfoton: transition 275 → 89; chlorpyrifos: transition 349 → 97).

### 3.3 Evaluation of the Adsorption Capacity of Malathion, Disulfoton, and Chlorpyrifos by Biosorbents

The adsorption efficiency of malathion, disulfoton, and chlorpyrifos was evaluated for eight biosorbents, including untreated grounds and husks as well as those subjected to acid, base, and carbonization treatments. The criterion for selection was the biosorbent exhibiting the highest multiple response values for the target pesticides calculated according to eq 1. Acid-pretreated coffee grounds were identified as the most effective sorbent.

It is important to emphasize that the acid-treated spent coffee grounds were not intended for long-term retention or remediation of pesticides. Rather, the material was developed as a renewable and low-impact sorbent for solid-phase extraction in the analytical sample preparation. During the SPE process, the analytes are temporarily retained on the biosorbent and subsequently eluted and quantified by LC–MS/MS, meaning that the pesticides do not remain bound to the material after extraction. Thus, the function of the biosorbent in this workflow is analytical, serving as a transient extraction phase rather than an environmental removal agent.

This clarification is relevant from a sustainability perspective. Traditional commercial sorbents commonly used in SPE procedures are often produced through chemically intensive or energy-demanding synthetic routes, which increase the cost and environmental impact. In contrast, the upcycling of spent coffee grounds, an abundant agro-industrial residue, through a simple acid-activation step provides a sustainable alternative that reduces resource consumption and waste generation while maintaining a suitable analytical performance. This approach aligns with the principles of Green Analytical Chemistry and circular resource use, reinforcing the environmental relevance of the proposed method.

### 3.4 pH-Dependent Zeta Potential Analysis and Isoelectric Point Determination

A pH-dependent zeta potential analysis was conducted to evaluate the surface charge behavior of the material, and the isoelectric point (IEP) was determined as the pH at which the zeta potential approached zero. The IEP of ASCG was approximately 3.6, as shown in Figure S2. This value reflects the predominance of acidic functional groups, primarily carboxylic (−COOH), whose dissociation p*K*_a_ typically falls within this pH range. Infrared analysis confirmed that acidified coffee grounds predominantly contain protonated carboxylic groups between pH 3.0 and 4.0, which accounts for the near-zero zeta potential observed at the IEP due to balanced surface charges. At this point of zero net surface charge, electrokinetic mobility is minimized, indicating equilibrium between positively and negatively charged sites on the material’s surface.

This surface behavior directly influences the extraction of malathion, disulfoton, and chlorpyrifos, none of which possess ionizable functional groups within the pH range investigated and therefore remain essentially neutral in aqueous solution, as summarized in Table S3 of the Supporting Information, which outlines their physicochemical properties. By adjustment of both the sample and the conditioning medium to pH 3.0, close to the isoelectric point (IEP) of the coffee grounds, the surface charge of the sorbent is minimized, reducing electrostatic repulsion toward hydrophobic or weakly ionized analytes. Under these conditions, nonelectrostatic interactions such as hydrophobic interactions, van der Waals forces, and potential hydrogen bonding become predominant, enhancing the affinity between the pesticides and the sorbent and consequently improving extraction efficiency.

### 3.5 Adsorption Kinetics and Isotherms

The experimental adsorption-kinetic data were fitted to pseudo-first-order, pseudo-second-order, chemisorption (Elovich), and fractional-order models, using the coefficients of determination (*R* ^2^) and the error function (*F*_error_) as evaluation criteria. A higher *R* ^2^ value (approaching 1.000) indicates a closer fit to the regression line, while lower *F*_error_ values denote a better model fit (Figure 5). The adsorption kinetics for malathion, disulfoton, and chlorpyrifos exhibited similar trends, with the pseudo-first-order model providing the best overall performance. For malathion, the pseudo-first-order model yielded an *R* ^2^ of 0.997 and the lowest *F*_error_ (2.651) among all models tested. Although the fractional-order model produced a slightly higher *R* ^2^ (0.999), its substantially larger error limited its predictive accuracy despite capturing the overall trend. For disulfoton, the pseudo-first-order model achieved an *R* ^2^ of 1.000 and an *F*_error_ of 0.633, indicating an excellent fit and minimal error (Tables S4–S6 in the Supporting Information). Similarly, for chlorpyrifos, the pseudo-first-order model resulted in an *R* ^2^ of 0.999 and an *F*_error_ of 1.077, confirming its superior performance among the models evaluated. These results suggest that pesticide adsorption is primarily governed by the availability of free adsorption sites, indicative of physisorption-dominated kinetics with a secondary contribution from chemisorption interactions.

**Figure 5:**
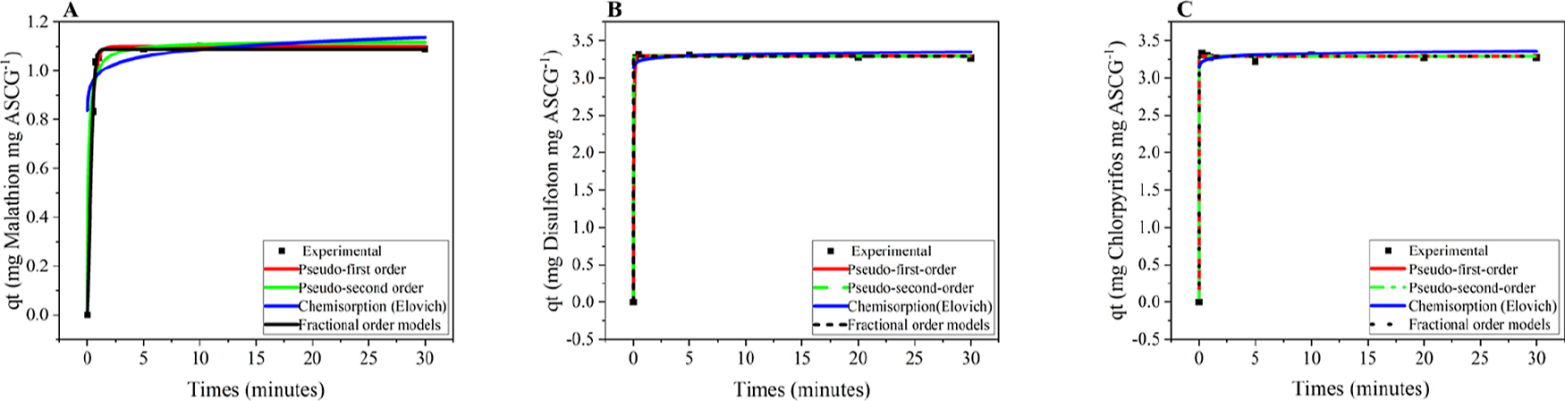
Adjusted kinetic models for (A) malathion, (B) disulfoton, and (C) chlorpyrifos.

In the isotherm study (Figure 6), the adsorption behavior of malathion was best described by the Langmuir model, which exhibited the lowest error (*F*_error_ = 30.859) and a satisfactory coefficient of determination (*R* ^2^ = 0.954). Although the Sips model provided a higher *R* ^2^ (0.985), it was associated with a substantially greater error, indicating a less reliable fit (Tables S7–S9 in the Supporting Information). These results suggest that malathion is predominantly adsorbed as a monolayer on a relatively homogeneous surface with minimal influence from surface heterogeneity.

**Figure 6:**
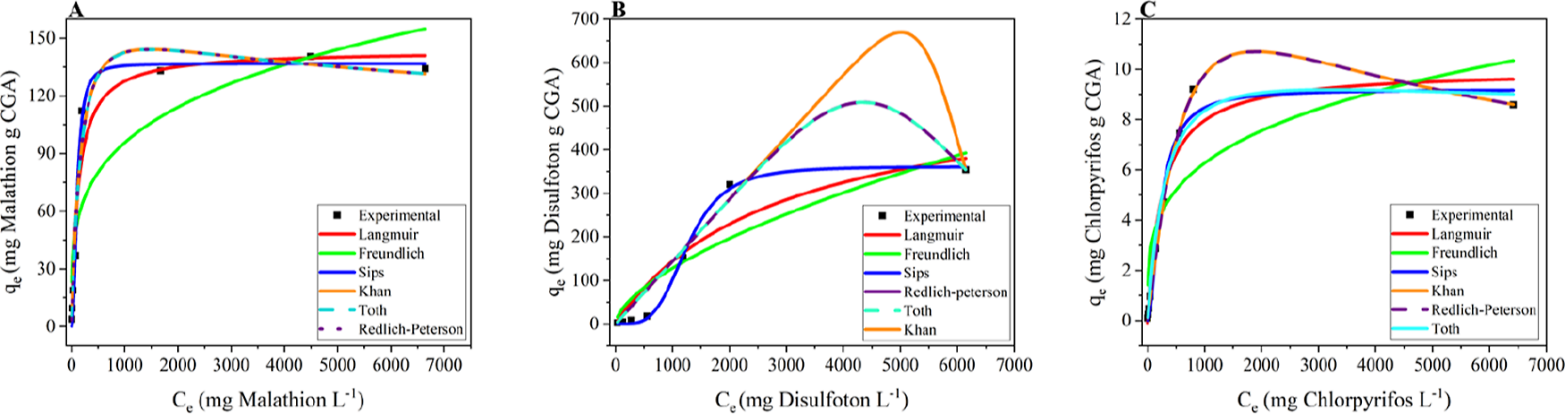
Adjusted isotherm models for (A) malathion, (B) disulfoton, and (C) chlorpyrifos.

The adsorption isotherms fitted with the Langmuir, Freundlich, Sips, and Redlich–Peterson models exhibited very similar curve profiles (Figure 6). This similarity arises from the mathematical and conceptual relationships among these models. The Sips and Redlich–Peterson equations are hybrid formulations that combine features of both Langmuir and Freundlich isotherms, behaving similarly to the Langmuir model at higher concentrations and to the Freundlich model at lower concentrations. Within the concentration range evaluated in this study, these hybrid models tend to generate overlapping curve shapes, often producing statistically equivalent fits. Moreover, when the empirical exponents in the hybrid models approach unity, their mathematical expressions converge toward the Langmuir equation, further reinforcing the resemblance observed among the isotherms. This convergence is consistent with the partially heterogeneous nature of the biosorbent surface and the limited concentration range investigated, conditions under which different empirical models can adequately describe the same adsorption phenomenon.^49,50^

For disulfoton, the Khan model provided the best representation of the data, with an *R* ^2^ of 0.935 and the lowest error among the models tested (*F*_error_ = 63.986), as well as a high maximum adsorption capacity, reflecting a strong affinity between the adsorbent and this compound. Although the Sips model yielded a higher *R* ^2^ value, its parameters were physically inconsistent, precluding its practical application. The Khan isotherm, a generalization of classical adsorption models, describes equilibrium in pure solutions by accommodating deviations from traditional models (Langmuir and Freundlich) and offering greater flexibility to represent diverse adsorption behaviors and surface mechanisms. The Khan isotherm posits that the amount adsorbed at equilibrium is a function of solute concentration, maximum saturation capacity, and adjustable parameters that allow for flexible representation of different adsorption mechanisms and surface heterogeneity.

For chlorpyrifos, the Khan model again provided the best balance between the goodness of fit (*R* ^2^ = 0.997) and accuracy (*F*_error_ = 52.245), outperforming the Langmuir model, which exhibited a considerably higher error and lower *R* ^2^.

In summary, the combined analysis of kinetic and isotherm models indicates that malathion adsorption follows pseudo-first-order kinetics and fits the Langmuir isotherm, suggesting a predominance of physical interactions and monolayer formation. In contrast, the adsorption of disulfoton and chlorpyrifos is described by pseudo-first-order kinetics and the Khan isotherm, also indicating primarily physical interactions.

### 3.6 Optimization of the Extraction Conditions

To optimize the extraction process, a fractional factorial design (2^5–1^) was used to investigate five variables: conditioning pH, sample pH, sorbent mass, sample volume, and eluent volume. This design enabled the evaluation of the main effects and interactions to identify significant variables.

A total of 19 experiments were conducted, including three replicates at the central point. The multiple response, calculated according to eq 1, served as the dependent variable. The results are presented in Table S10 in the Supporting Information.

The significance of the main effects and interactions was assessed at the 95% confidence level using a Pareto chart, where columns represent the magnitude of each contrast, and the red vertical line indicates statistical significance at *p* = 0.05.^51^ The Pareto chart corresponding to Figure S3 in the Supporting Information was generated using Statistica software.

According to Pareto analysis, none of the variables or their interactions were statistically significant within the experimental domain evaluated. Consequently, all factors were set at their minimum levels except for sample volume. The optimized extraction conditions were: conditioning pH 3.0, sample pH 3.0, adsorbent mass 25.0 mg, sample volume 10 mL, and eluent volume 0.5 mL. The selection of pH 3.0 for both conditioning and sample preparation is justified using formic acid, which is commonly employed in mass spectrometry to enhance analyte ionization.^52^ The increased sample volume was chosen due to the abundance and ease of collecting the matrix, as well as the potential to improve the preconcentration factor. Figure 7 shows a representative chromatogram of a sample spiked with malathion, disulfoton, and chlorpyrifos at 1.0 mg L^–1^ under the optimized extraction conditions.

**Figure 7:**
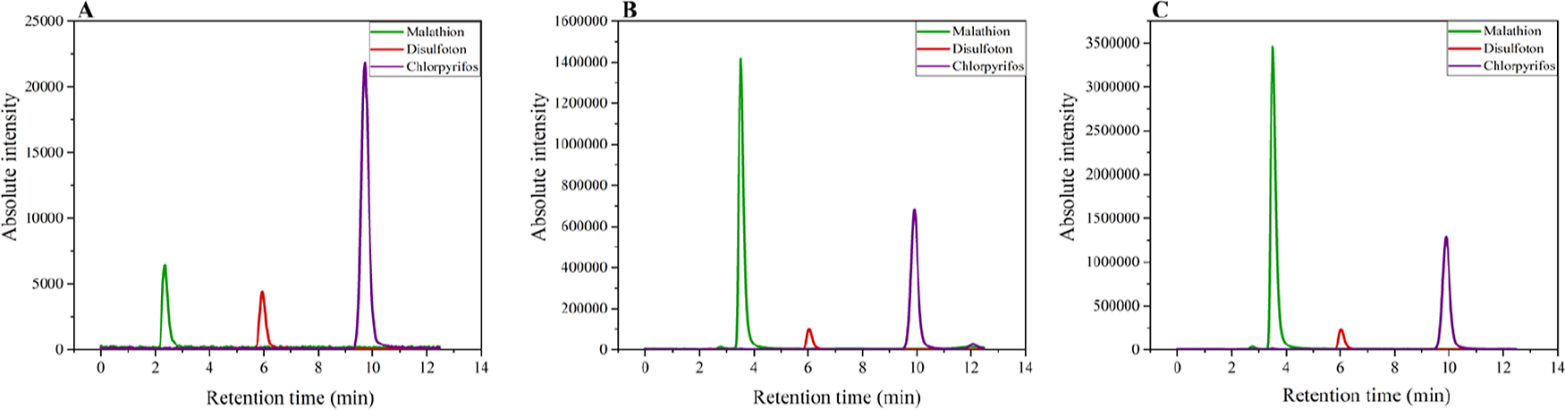
Chromatograms of extracts from samples spiked with malathion (green, RT = 3.5 min, transition 331 → 127), disulfoton (red, RT = 6.0 min, transition 275 → 89), and chlorpyrifos (purple, RT = 9.8 min, transition 349 → 97) under optimized chromatographic conditions. From left to right: (A) 5.0 μg L^–1^ malathion, 25.0 μg L^–1^ disulfoton and chlorpyrifos; (B) 100.0 μg L^–1^ all compounds; and (C) 250. μg L^–1^ all compounds.

### 3.7 Evaluation of the Cleanup Step in the Extraction Process

The extraction of five samples spiked with malathion, chlorpyrifos, and disulfoton at a concentration of 1.0 mg L^–1^, as well as an analytical blank, was performed following the sample analyses. To assess the efficiency and necessity of the cleanup step, selected reaction monitoring (SRM) transitions of caffeine (195 → 138), a compound inherently present in coffee grounds, were also monitored. Coefficient of variation (% CV) values below 15.0% for all analytes indicate excellent repeatability, confirming the reliability of the analytical method employed.

However, the significant detection of caffeine in the replicates, even after the cleanup process, highlights the inefficiency of this step in completely removing this compound, raising questions about its necessity in the developed method. The inclusion of the washing step did not result in a significant improvement in analyte recoveries and increased only the complexity and duration of the analyses. Therefore, the washing step was omitted from the extraction procedure. Nonetheless, the observed memory effect confirms the need for an intermediate cleaning step between extractions to prevent carryover.

### 3.8 Evaluation of the Memory Effect

To assess the efficacy of cartridge cleanup between extractions and the feasibility of cartridge reuse, thereby eliminating memory effects, 1.00 mL of methanol acidified with 0.1% formic acid was passed through the cartridge between each extraction. Ten consecutive extractions were performed using the same cartridge, and the coefficients of variation (CV) for all three target analytes were below 15.0% (malathion: 14.8%; disulfoton: 12.5%; chlorpyrifos: 14.0%). The blank sample, analyzed after extraction of the spiked matrix, showed no detectable peaks at the retention times of the analytes.

### 3.9 Evaluation of the Matrix Effect

To assess the presence of matrix effects in the developed analytical method, the procedure recommended by SANTE/11312/2021 was used. Qualitative evaluation was performed by comparing the slopes of calibration curves prepared in methanol and matrix extract. According to the guidelines, a matrix effect is confirmed when the calibration curves do not overlap.

As illustrated in Figure 8, the matrix effect was observed in the proposed method for the determination of malathion, disulfoton, and chlorpyrifos in environmental water samples, as indicated by the distinct slopes of the calibration curves. The green curve represents malathion, the red curve disulfoton, and the purple curve chlorpyrifos.

**Figure 8:**
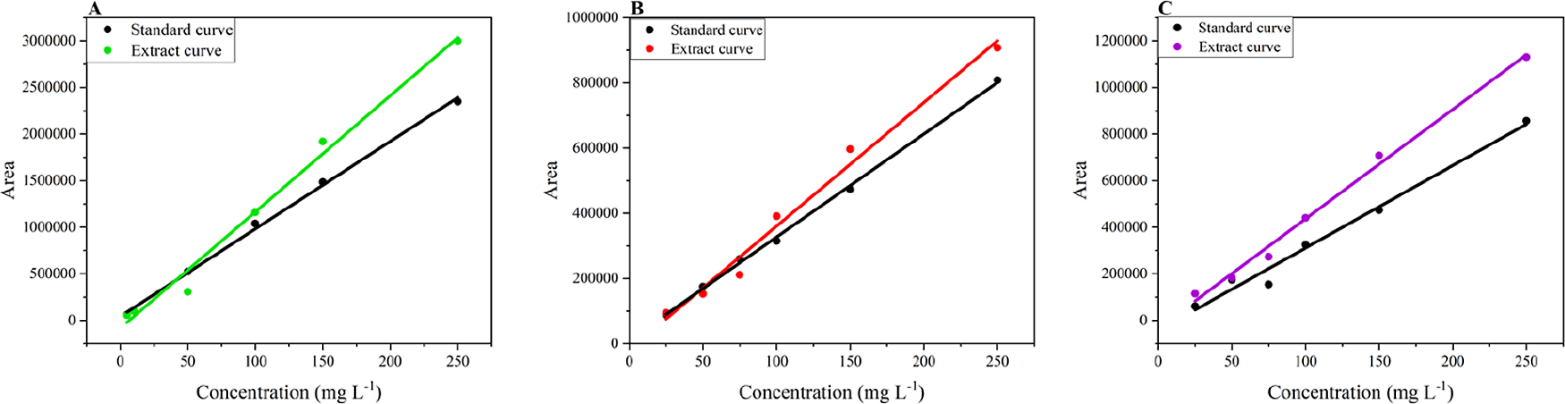
Matrix effect evaluation, comparison between standard calibration curves (black) and extract matrix curves for three pesticides. (A) Malathion (green), concentration range 5.0 to 250.0 μg L^–1^. (B) Disulfoton (red), concentration range 25.0 to 250.0 μg L^–1^. (C) Chlorpyrifos (purple), concentration range 25.0 to 250.0 μg L^–1^. The matrix curves show differences in response compared to the standard curves, indicating matrix effects in the sample extracts.

In addition to the qualitative assessment, a quantitative evaluation of the matrix effect was conducted by calculating the percentage of matrix effect (% ME) as described in eq 4. Using the slopes of the calibration curves prepared in methanol and matrix extract, % ME values of 32.8% for malathion, 19.9% for disulfoton, and 32.9% for chlorpyrifos were obtained. According to Economou et al.,[Bibr ref53] (2009), these results indicate a moderate matrix effect for malathion and chlorpyrifos, and a negligible effect for disulfoton. Established criteria classify variations below 20.0% as negligible, between 20.0% and 50.0% as moderate, and above 50.0% as strong matrix effects.

Given the moderate matrix effect observed for two of the pesticides, quantification should be performed using the calibration curve constructed in the matrix extract to adequately compensate for this effect. It is important to note that matrix effects are commonly reported in analyses involving complex matrices.^54^

### 3.10 Figures of Merit

The method demonstrated a linear detection range of 5.0–250.0 μg L^–1^ for malathion and 25.0–250.0 μg L^–1^ for disulfoton and chlorpyrifos, with coefficients of determination (*R* ^2^) of 0.995 for malathion, 0.996 for disulfoton, and 0.995 for chlorpyrifos.

The linear regression equations were as follows:

Peak area = 962,850 [malathion] + 460,020; Peak area = 9535.9 [disulfoton] + 178,096; Peak area = 36,425 [chlorpyrifos] + 322,194.

The precision and accuracy of the method were evaluated for each pesticide individually. For malathion, intraday precision showed coefficient of variation (CV %) values ranging from 13.7% to 15.0%, while interday precision varied from 10.0% to 12.0%. Recovery values ranged from 97.1% to 107.1% in the intraday analysis and from 93.1% to 95.3% over 3 days, demonstrating acceptable precision and accuracy.

In the case of disulfoton, the method exhibited excellent intraday precision, with CV % values ranging from 1.4% to 6.9%, and interday CVs between 8.4% and 8.9%. Recovery rates ranged from 85.9% to 109.3% for intraday measurements and from 86.9% to 102.0% across 3 days, indicating consistent analytical performance and reliable quantification over time.

For chlorpyrifos, intraday precision showed CVs ranging from 4.1% to 4.5%, and interday precision ranged from 7.7% to 8.7%, with CVs up to 11.1%. Recovery values were within 97.0% to 99.9% in intraday evaluations and between 92.6% and 100.5% in the interday analysis, confirming both the accuracy and reproducibility of the method for this analyte.

The limits of detection and quantification were determined experimentally by progressively decreasing the analyte concentration in the matrix until the respective limits were reached. The LOD and LOQ for malathion were 1.67 μg L^–1^ and 5.0 μg L^–1^, respectively, and for disulfoton and chlorpyrifos, 8.33 μg L^–1^ and 25.0 μg L^–1^, respectively. Table 2 summarizes the analytical performance data.

**Table 2:** Analytical Performance Parameters

parameters	malathion	disulfoton	chlorpyrifos
linear range (μg L^–1^)	5.0–250.0	25.0–250.0	25.0–250.0
equation	*y* = 96285*x* + 460020	*y* = 9535.9*x* + 178096	*y* = 36425*x* + 322194
coefficient of determination (*R* ^2^)	0.995	0.996	0.995
correlation coefficient	0.997	0.998	0.997
LOD (μg L^–1^)	1.67	8.33	8.33
LOQ (μg L^–1^)	5.0	25.0	25.0
intraday precision CV %	12.6^a^	3.6^b^	8.3^b^
	13.7^c^	1.4^c^	4.5^c^
	15.0^d^	6.9^b^	4.1^d^
interday precision CV %	12.0^a^	6.6^b^	8.7^b^
	10.0^c^	8.4^c^	11.1^c^
	10.0^d^	8.9^d^	7.7^d^
recovery (%) (*n* = 6)	97.1^a^	85.9^b^	97.9^b^
	107.1^c^	109.3^c^	97.1^c^
	97.3^d^	99.6^d^	99.9^d^
recovery (%) (*n* = 6, 3 days)	94.0^a^	86.9^b^	92.6^a^
	93.1^c^	93.1^c^	102.0^c^
	95.3^d^	102.0^d^	100.5^d^

a 5.0 μg L^–1^.

b 25.0 μg L^–1^.

c 100.0 μg L^–1^.

d 250.0 μg L^–1^.

Table 3 shows a comparison between this study and methods previously reported in the literature, highlighting the analytical technique, sample preparation techniques, LOQ, and linear range.

**Table 3:** Comparison Between Studies Reported in the Literature and Those Obtained in This Work for the Determination of Organophosphates in Water Samples

pesticides	sample preparation technique	LOQ (μg L^–1^)	linear range (μg L^–1^)	reference
malathion	SPE with polymeric sorbent	0.5	10–250	^55^
	hollow fiber liquid-phase microextraction (HF-LPME)	0.00341	0.05–10.00	^56^
	MIP	0.03	0.0005–0.1	^57^
	MSU-1-UPLC-MS/MS	0.25	5.00–100.00	^58^
	ionic liquid dispersive liquid liquid microextraction (IL-DLLME)	2.5	2.50–50.00	^59^
	SPE	5	5–250	current Study
disulfoton	hollow fiber liquid-phase microextraction (HF-LPME)	0.01125	0.05–100	^60^
	SPE	25 μg L^–1^	25–250 μg L^–1^	current Study
chlorpyrifos	SPE with polymeric sorbent	0.5	10–250	^61^
	hollow fiber liquid-phase microextraction (HF-LPME)	0.325	0.10–10.00	^62^
	SPE	0.026	0.0005–0.1	^63^
	SPE	25.0 μg L^–1^	25–250 μg L^–1^	current Study

The comparison between the method developed in this study and those previously reported in the literature highlights clear advances in both analytical efficiency and sustainability. In this work, only 25 mg of acid-treated coffee grounds and 10 mL of sample were required to establish a low-cost, simple, and environmentally friendly SPE system that eliminates the need for synthetic sorbents.

When compared with the method of Donato et al. (2015),^55^ which required 60 mg of polymeric C18 sorbent and 100 mL of sample, the approach developed in this study achieved a substantial reduction in both sorbent mass and sample volume while maintaining satisfactory analytical performance (linear range: 10–250 μg L^–1^; recoveries >85%). Moreover, whereas Donato et al.^55^ defined the LOQ based on a signal-to-noise ratio of 10, the present study adopted the lowest calibration point as the LOQ. Although this results in numerically higher values, it provides a more robust and representative quantification limit by avoiding uncertainties associated with noise-level signals.

Similarly, the method reported by Rocha et al. (2015)^57^ relied on C18 cartridges containing 500 mg of sorbent and 200 mL of sample—conditions associated with higher costs, elevated solvent consumption, and increased waste generation. In contrast, the coffee-based biosorbent used here reduced the sorbent mass by approximately 20-fold and sample volume by 95%, while still ensuring efficient extraction of malathion, disulfoton, and chlorpyrifos. Additionally, coffee residues are renewable and abundant agro-industrial byproducts, further reinforcing their sustainable advantage over nonrecyclable synthetic sorbents.

The method described by Kharbouche et al. (2019),^58^ which employed MSU-1 sorbents coupled with UPLC-MS/MS, achieved very low LOQs (0.25 μg L^–1^) using EURACHEM-based criteria (S/N = 10). However, it relied on high-cost synthesized mesoporous materials and labor-intensive preparation procedures. Although the LOQs obtained in this study are higher (5–25 μg L^–1^), the proposed method is simpler, more economical, more environmentally friendly, and better suited for routine applications, offering practical advantages that complement analytical performance.

The IL-DLLME method proposed by Marube et al. (2018)^59^ achieved an LOQ of 2.5 μg L^–1^ for malathion but featured a narrow linear range (2.5–50 μg L^–1^) and required toxic, expensive, and poorly biodegradable ionic liquids. The coffee-based SPE method avoids hazardous solvents and provides a wider analytical range, good precision (CV ≤15%), and high reproducibility, effectively combining sustainability with analytical reliability.

In the study by Salvatierra-Stamp et al. (2021),^56^ LOQs were calculated using the expression (10 × s)/b. Although sensitive, this criterion may underestimate true quantification limits due to its strong dependence on instrumental stability and baseline noise. Furthermore, the method required nonreusable polymeric fibers, 1 L of sample, and 1-octanol, resulting in greater cost, operational complexity, and waste generation. In comparison, the coffee-biosorbent strategy presented here is considerably simpler and more sustainable.

It is important to emphasize that validated methods for determining pesticides in environmental waters remain limited, particularly for disulfoton, which further underscores the relevance of developing sensitive and sustainable approaches for monitoring this compound.

Overall, the method developed in this study represents a meaningful improvement over existing methodologies by integrating a reliable analytical performance, more realistic LOQ criteria, and strong environmental advantages. Although defining the LOQ as the lowest calibration point yields numerically higher values than noise-based methods, it offers greater reproducibility and practical relevance. Acid-treated coffee grounds, therefore, emerge as an efficient, cost-effective, and green biosorbent capable of replacing high-cost synthetic materials without compromising analytical quality, thereby promoting more accessible and sustainable laboratory practices.

Finally, it is noteworthy that the acid-pretreated coffee sorbent is reusable. A washing test involving ten sequential rinses with 1.00 mL of methanol acidified with 0.1% formic acid demonstrated no loss of performance. After each wash, malathion, disulfoton, and chlorpyrifos were quantified in aqueous standards at 100 μg L^–1^, and CV values remained below 15% for all analytes, confirming the robustness and reusability of the biosorbent.

### 3.11 Application of the Proposed Method

Real environmental water samples (groundwater and surface water) were analyzed under optimized and validated conditions to evaluate the applicability of the method. Initially, the pH of the samples was adjusted, and then the pesticide analysis was performed using the proposed method. Fourteen samples were collected in the region of Alfenas, in the state of Minas Gerais (MG), Brazil, and four samples presented traces of organophosphate pesticides, as indicated in Table 4.

**Table 4:** Levels of Malathion, Disulfoton, and Chlorpyrifos Found in Drinking and Environmental Water Using the Developed Method

samples	concentration (μg L^–1^)	CV (%)
1	^a^not detected	
	^b^not detected	
	^c^not detected	
2	^a^not detected	
	^b^not detected	
	^c^not detected	
3	^a^not detected	
	^b^not detected	
	^c^not detected	
4	^a^not detected	
	^b^not detected	
	^c^<LOQ	
5	^a^<LOQ	
	^b^not detected	
	^c^<LOQ	
6	^a^<LOQ	
	^b^not detected	
	^c^not detected	
7	^a^not detected	
	^b^not detected	
	^c^<LOQ	
8	^a^not detected	
	^b^not detected	
	^c^not detected	
9	^a^not detected	
	^b^not detected	
	^c^not detected	
10	^a^not detected	
	^b^33.03	7.71
	^c^not detected	
11	^a^not detected	
	^b^not detected	
	^c^not detected	
12	^a^not detected	
	^b^76.75	9.37
	^c^not detected	
13	^a^not detected	
	^b^69.66	0.62
	^c^not detected	
14	^a^not detected	
	^b^not detected	
	^c^not detected	

a Malathion.

b Disulfoton.

c Chlorpyrifos.

Two samples of malathion and three samples of chlorpyrifos were found to be below the limit of quantification (<LOQ). Additionally, three samples showed disulfoton levels above the limit of quantification (>LOQ) at concentrations of 33.03, 76.75, and 69.66 μg L^–1^, respectively, which fall within the maximum residue limits established by the World Health Organization (WHO). The WHO recommends a maximum permitted value in water of 100 μg L^–1^ for each pesticide and 500 μg L^–1^ for the total pesticide content.^64^ The detection of organophosphate pesticide residues, such as malathion, disulfoton, and chlorpyrifos, in environmental water samples highlights the crucial importance of developing and implementing sensitive and specific analytical methods for monitoring these contaminants. Efficient and continuous monitoring of pesticide levels is essential not only to ensure compliance with regulatory limits but also, above all, to safeguard consumer health and protect the integrity of aquatic ecosystems. Considering the known toxicological risks of these compounds including neurotoxic effects, developmental disorders, and serious environmental impacts the modernization of analytical techniques contributes to the rapid identification of contamination and the adoption of effective remediation measures, reinforcing the commitment to public health and environmental sustainability.^65^

### 3.12 Green Chemistry Metrics

Green Analytical Chemistry (GAC) seeks to reduce the environmental footprint of chemical analyses by addressing key concerns, such as the production of toxic laboratory waste and the utilization of solvents and reagents that are harmful to both human health and the environment. Established nearly a decade ago, the 12 principles of GAC provide a comprehensive framework to guide the development and implementation of more sustainable analytical practices.

In the present investigation, GAC parameters were systematically assessed using one widely recognized “green analytical methods”: AGREEPREP. The AGREEPREP methodology employs ten distinct impact categories, each converted into a subscore on a scale of 0–1. These subscores are then integrated to yield a final assessment value. The evaluation criteria encompass several critical aspects, including solvent, material, and reagent selection; waste generation; energy consumption; sample size; and yield. The pictogram generated by the AGREEPREP analysis indicated a sustainability score of 0.64, demonstrating that the method achieves an acceptable level of compliance with the principles of green chemistry and exhibits a more environmentally friendly profile compared to conventional sample-preparation procedures (Figure 9).

**Figure 9:**
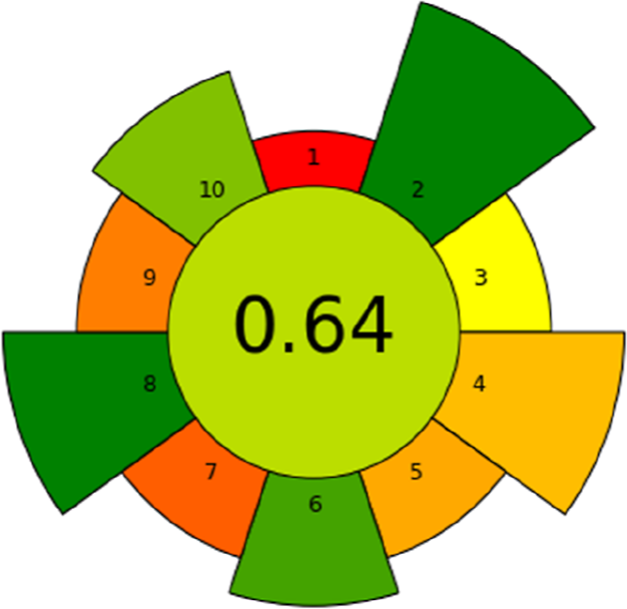
Analysis of green chemistry-related metrics.

When comparing our results with the study conducted by Wojnowski et al. (2022),^66^ who evaluated different sample preparation techniques such as LLE and SPE using MIPs, a much lower score of 0.17 was obtained due to the high solvent consumption and the greater number of operational steps. In the same study, SPME and online SPE achieved scores of 0.55 and 0.54, respectively. The SPE method developed in this study reached a score of 0.64, surpassing all methodologies evaluated by those authors and indicating a balanced combination of selectivity, operational simplicity, lower environmental impact, and operator safety.

## 4 Conclusions

This study demonstrated the potential of upcycled coffee waste as an efficient and sustainable biosorbent for the extraction of organophosphorus pesticides from environmental waters. Among the eight materials derived from coffee husks and spent coffee grounds subjected to different chemical and thermal treatments, acid-treated spent coffee grounds exhibited the best adsorption and extraction performance. The comprehensive characterization confirmed that surface functionalization with carboxylic groups enhanced interactions with the analytes, particularly near the material’s isoelectric point (pH 3.6), favoring predominantly nonelectrostatic adsorption mechanisms.

The optimized SPE–LC–MS/MS method showed excellent analytical performance. Using a fractional factorial design, the extraction conditions were refined to maximize recoveries of malathion, disulfoton, and chlorpyrifos, while minimizing experimental effort, solvent consumption, and adsorbent use. The method provided wide linear ranges (5.0–250.0 μg L^–1^ for malathion and 25.0–250.0 μg L^–1^ for disulfoton and chlorpyrifos), high correlation coefficients, satisfactory precision and accuracy, and low limits of detection and quantification (LOD: 1.67 μg L^–1^ for malathion; 8.33 μg L^–1^ for disulfoton and chlorpyrifos). The AGREEPREP evaluation yielded a sustainability score of 0.64, indicating substantial alignment with Green Analytical Chemistry principles and highlighting the environmental benefits of repurposing agricultural waste.

Application of the method to 14 environmental water samples from the Alfenas–MG region resulted in the detection of pesticides in four samples, with quantification in three of them. These results underscore both the practical applicability of the method and the ongoing need for reliable monitoring tools to support the environmental surveillance of pesticide contamination. Overall, the findings demonstrate that coffee waste is a promising green sorbent capable of supporting more sustainable analytical practices.

Nevertheless, some limitations should be acknowledged. The study investigated a limited set of real samples and focused exclusively on three organophosphorus pesticides, which restricted the generalization of the method to broader contaminant classes. Additionally, performance was not evaluated in more complex matrices nor compared to alternative green extraction techniques. Future work should expand the range of analytes, assess diverse environmental conditions, and explore large-scale or automated applicability to further validate the analytical robustness and environmental impact of this sustainable approach.

## Supplementary Material



## Notes

Material characteristics obtained using the BET equation, including specific surface area, pore volume, and pore size distribution; nitrogen adsorption–desorption isotherms and the corresponding pore size distribution curves; optimized MS/MS parameters for the determination of malathion, disulfoton, and chlorpyrifos; zeta potential measurements of acid-pretreated coffee grounds as a function of pH; physicochemical properties of the pesticides evaluated in this study; models, equations, and estimated parameters for the kinetic studies of malathion, disulfoton, and chlorpyrifos; models, equations, and parameters estimated for the adsorption isotherms of each pesticide; fractional factorial design used in the optimization experiments; and Pareto chart generated from the 2^5–1^ fractional factorial design (PDF)
