# Supplementary material for: Upcycled Coffee Waste as Sustainable Sorbents for Monitoring Organophosphorus Pesticides in Environmental Waters
Source: ACS Omega. 2025 Dec 22;11(1):1982–98. doi: 10.1021/acsomega.5c10460 (PMC12809546; doi:10.1021/acsomega.5c10460)
Supplement: Supplementary file 1 [file ao5c10460_si_001.pdf]

Upcycled Coffee Waste as Sustainable Sorbents for Monitoring Organophosphorus Pesticides in Environmental Waters

Saulo Alves de Souza[a], Gabriel Oliveira Araújo Costa[a], Grazielle Cabral de Lima[a], Cristiane Dos Reis Feliciano[a], Rudy Bonfilio[b], Mariane Gonçalves Santos[a*]

[a] *Instrumental Analytical Chemistry Research Group – GPQAI, Institute of Chemistry, Federal University of Alfenas - Unifal-MG, Alfenas, MG, 37130-001, Brazil.*

[b] *Faculty of Pharmaceutical Sciences, Federal University of Alfenas - UNIFAL-MG, Alfenas, MG, 37130-001, Brazil.*

*Address correspondence to Mariane Gonçalves Santos, Instrumental Analytical Chemistry Research Group, Institute of Chemistry, Federal University of Alfenas, Alfenas, Minas Gerais, 37130-001, Brazil. E-mail: mariane.goncalves@unifal-mg.edu.br.

Syntheses and characterizations of biosorbents

The results obtained from the BET analysis studies of the materials are presented in Table S1 and Figure S1.

Material	Specific surface area (m^2 g^{-1})	Pore size (cm3 g-1)	Pore diameter (nm)
CH	2.436	0.001063	1.83
CCH	11.434	0.00456	2.88
ACH	0.8953	0.001237	5.03
BCH	0.8349	0.001293	3.93
SCG	0.9793	0.000494	1.54
CSCG	175.6881	0.134979	11.27
ASCG	1.592	0.000838	1.52
BSCG	1.1058	0.000748	1.86

Table S1. Material characteristics, such as specific surface area, pore volume, and pore size distribution, obtained through the application of the BET (Brunauer-Emmett-Teller) equation.

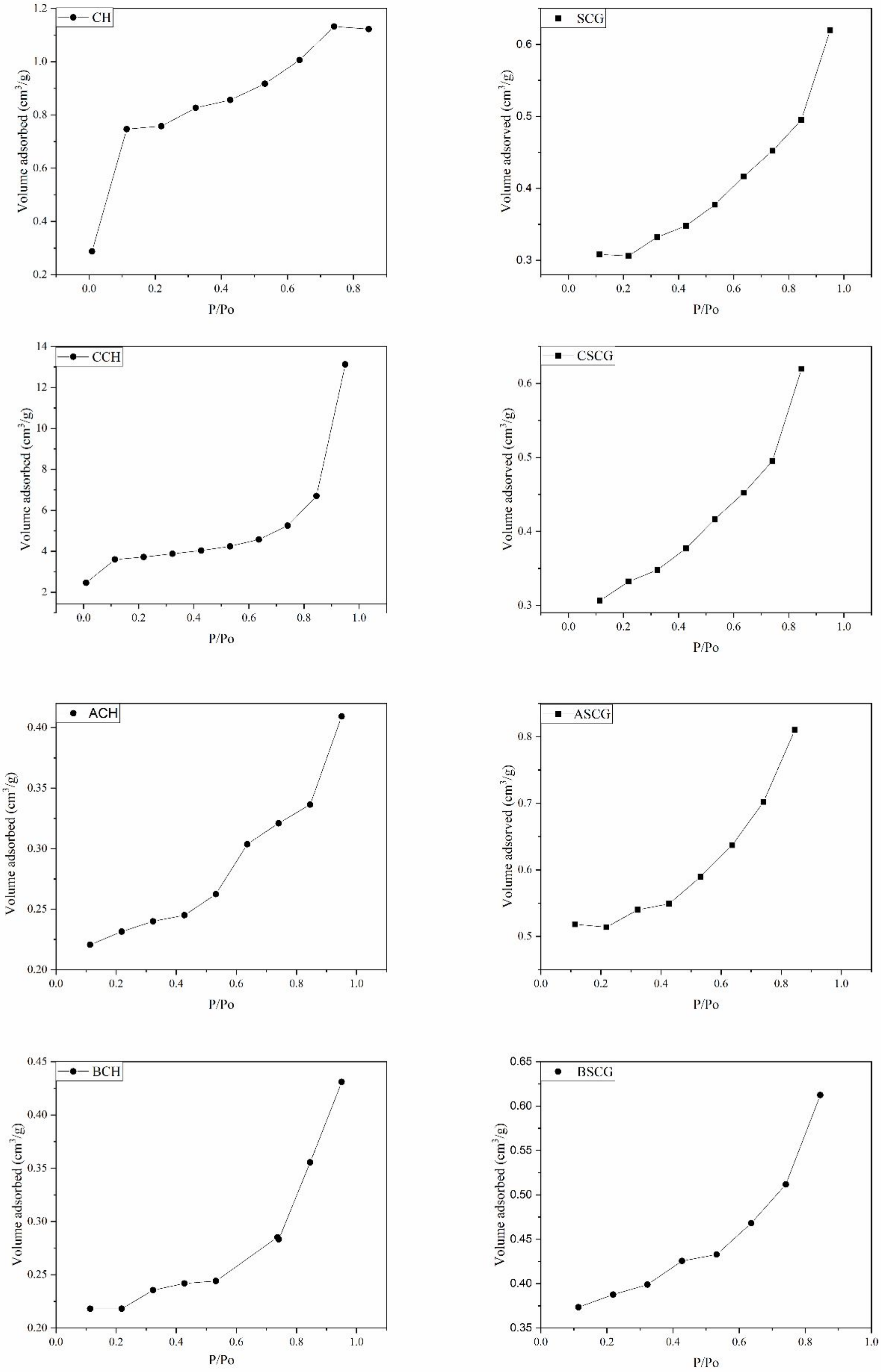

CH
Volume adsorbed (cm^3/g)
P/Po
SCG
Volume adsorved (cm^3/g)
P/Po
CCH
Volume adsorbed (cm^3/g)
P/Po
CSCG
Volume adsorved (cm^3/g)
P/Po
ACH
Volume adsorbed (cm^3/g)
P/Po
ASCG
Volume adsorved (cm^3/g)
P/Po
BCH
Volume adsorbed (cm^3/g)
P/Po
BSCG
Volume adsorbed (cm^3/g)
P/Po

Figure S1. Nitrogen adsorption–desorption isotherms and pore size distribution curves for CH, SCG, CCH, CSCG, ACH, ASCG, BCH, and BSCG, showing the Type II, III, and IV profiles and the corresponding microporous–mesoporous characteristics of the materials.

Analyte	Precursor ion (m/z)	Fragmentation (m/z)	CE (eV)	Ionization mode
Malathion	331.0	127.0	-14.0	+
		98.9	-25.0	
		284.9	-9.0	
Disulfoton	275.0	89.0	-10.0	+
		73.9	-22.0	
		60.9	-35.0	
Chlorpyrifos	350.0	96.9	-32.0	+
		197.8	-22.0	
		124.9	-21.0	

Optimization of ms/ms conditions

Table S2. Optimization of MS/MS parameters for the determination of malathion, disulfoton, and chlorpyrifos.

pH-Dependent Zeta Potential Analysis and Isoelectric Point Determination

Variation of the zeta potential of acid-treated coffee grounds (CGA) as a function of pH, ranging from 3.0 to 10.0.

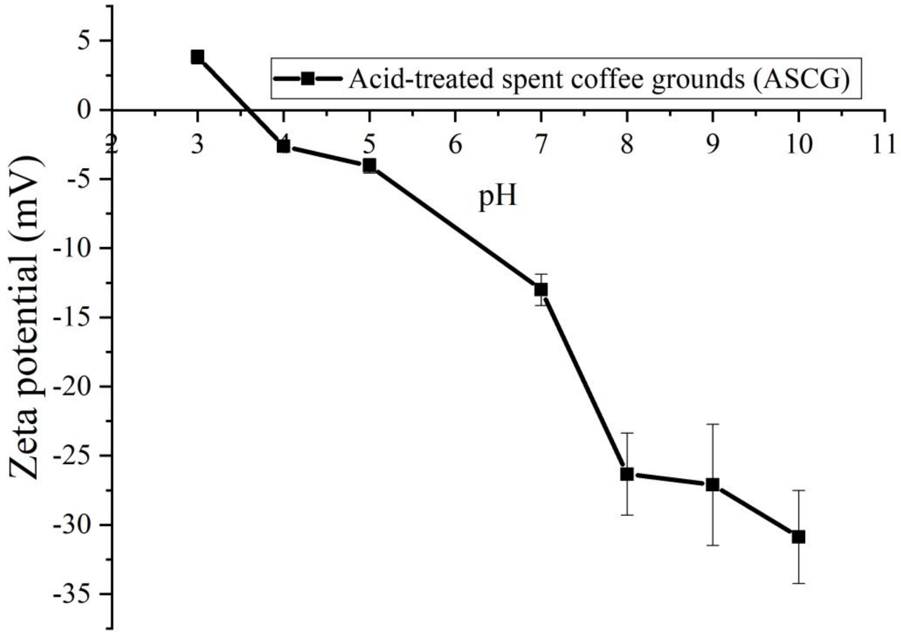

Figure S2. Zeta potential measurements of coffee grounds pre-treated with acid as a function of pH.

Table S3 summarizes the physicochemical properties of malathion, disulfoton, and chlorpyrifos, including their toxicological classification, log D, pKa, and chemical structure.

Table S3. Physical-chemical characteristics of the pesticides that were determined in samples in this study.

Name	Toxicological class	logD	pKa	Chemical structure
Malathion	Class 3	2.93	-	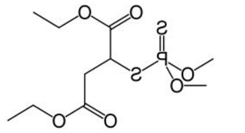
Disulfoton	Class 1	3.61	-	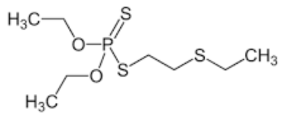
Chlorpyrifos	Class 2	-	-	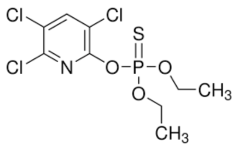

Class 1 - Extremely Toxic Products; Class 2 - Highly Toxic Products; Class 3 - Moderately Toxic Products; Class 4 - Slightly Toxic Products; Class 5 - Products Unlikely to Cause Acute Harm.

In this study, the physicochemical properties of three widely used organophosphate pesticides, malathion, disulfoton, and chlorpyrifos, were investigated, as show in the Table S3. Structural analysis indicated that both malathion and disulfoton lack ionizable groups in their molecular structures, resulting in constant log D values across the entire pH range studied (1–14). For malathion ($C_{10}H_{19}O_6PS_2$), the calculated log D was 2.93, while for disulfoton ($C_8H_{19}O_2PS_3$), a log D of 3.61 was obtained. The octanol–water partition coefficients (log P) were determined to be 2.93 ± 0.35 for malathion and 3.61 ± 0.22 for disulfoton, values consistent with the lipophilic nature of these organophosphorus compounds. All data were processed and organized using ACD/ChemSketch software.

For chlorpyrifos ($C_9H_{11}Cl_3NO_3PS$), the ChemSketch software was unable to calculate log D or pKa values due to the absence of identifiable ionizable groups within the pH range of 1–14. The calculated log P for chlorpyrifos was 4.77 ± 0.40, which aligns with literature values ranging from 4.7 to 5.01. It should be noted that, although prediction algorithms do not explicitly confirm the absence of ionizable groups in chlorpyrifos, theoretical analyses suggest that protonation of the nitrogen atom in the pyridine ring would only occur under extremely acidic conditions (pH < 0), a scenario rarely encountered in environmental or biological systems. These characteristics underscores the importance of experimental validation or consultation of specialized literature for definitive confirmation of the ionization properties of this organophosphate pesticide.

Adsorption kinetics and isotherms

The Table S4-S9 below present the models, equations, and estimated parameters for the kinetic models, namely, pseudo-first-order, pseudo-second-order, chemisorption (Elovich), and fractional-order models, as well as the models, equations, and estimated parameters for the isotherm models, including Langmuir, Freundlich, Sips, Khan, Toth, and Redlich–Peterson, applied to malathion, disulfoton, and chlorpyrifos.

Table S4. Models, equations, and estimated parameters for kinetic models - malathion.

kinetic models	Equation	Parameter	Data
Pseudo-first-order	$q_t = q_e[1 - \exp(-k_1\ t)]$	qe (mg g^{-1})	1.099
		k_1 (min^{-1})	3.094
		R^2	0.997
		F_{error}	2.651
Pseudo-second-order	$q_t = \frac{k_2\, q_e^2\ t}{1 + k_2\, q_e\, t}$	qe (mg g^{-1})	1.120
		K_2 (min^{-1})	7.747
		R^2	0.983
		F_{error}	5.520
Chemisorption (Elovich)	$q_t = \frac{1}{k}\ln(n\ k) + \frac{1}{k}\ln(t)$	N	4.18E8
		K (min^{-1})	23.265
		R^2	0.967
		F_{error}	8.053
Fractional order models	$q_t = q_e[1 - \exp(-k\ t)$	qe (mg g^{-1})	1.089
		K (min^{-1})	2.524
		N	1.625
		R^2	0.999
		F_{error}	69.016

Table S5. Models, equations, and estimated parameters for kinetic models - dissulfoton.

kinetic models	Equation	Parameter	Data
Pseudo-first-order	$q_t = q_e[1 - \exp(-k_1\ t)]$	qe (mg g^{-1})	3.292
		k_1 (min^{-1})	20.021
		R^2	1.000
		F_{error}	0.633
Pseudo-second-order	$q_t = \frac{k_2\, q_e^2\ t}{1 + k_2\, q_e\, t}$	qe (mg g^{-1})	3.289
		K_2 (min^{-1})	1108.950
		R^2	1.000
		F_{error}	0.687932
Chemisorption (Elovich)	$q_t = \frac{1}{k}\ln(n\, k) + \frac{1}{k}\ln(t)$	N	4.84E44
		K (min^{-1})	32.782
		R^2	0.997
		F_{error}	2.160
Fractional order models	$q_t = q_e[1 - \exp(-k\ t)\hat{}n]$	qe (mg g^{-1})	3.299
		K (min^{-1})	3.78E-4
		N	-0.335
		R^2	1.000
		F_{error}	107.915

Table S6. Models, equations, and estimated parameters for kinetic models - chlorpyrifos.

kinetic models	Equation	Parameter	Data
Pseudo-first order	$q_t = q_e[1 - \exp(-k_1\ t)]$	qe (mg g^{-1})	3.288
		k_1 (min^{-1})	6621.318
		R^2	0.999
		F_{error}	1.077
Pseudo-second order	$q_t = \frac{k_2\, q_e^2\ t}{1 + k_2\, q_e\, t}$	qe (mg g^{-1})	3.288
		K_2 (min^{-1})	-1.07E44
		R^2	0.999
		F_{error}	12.510
Chemisorption (Elovich)	$q_t = \frac{1}{k}\ln(n\,k) + \frac{1}{k}\ln(t)$	N	5.95E44
		K (min^{-1})	32.732
		R^2	0.995
		F_{error}	2.420
Fractional order models	$q_t = q_e[1 - \exp(-k\ t)\^n]$	qe (mg g^{-1})	3.288
		K (min^{-1})	142.064
		N	3.154
		R^2	0.999
		F_{error}	68.434

Table S7. Models, equations, and estimated parameters for isotherm models - malathion.

Isotherm model	Equation	Parameter	Data
Langmuir	$q_e = \frac{q_s K_l C_e}{1+bC_e}$	q_s (mg g^{-1})	143.526
		K_L (mg L^{-1})	0.008
		R^2	0.954
		F_{error}	30.859
Freundlich	$q_e = K_f C_e^{1/ns}$	K_F (mg.g^{-1}) (mg.L^{-1})^n	16.474
		n_F	3.928
		R^2	0.789
		F_{error}	229.062
Sips	$q_e = \frac{q_s K_s C_e^{1/ns}}{1 + a_s C_e^{1/ns}}$	q_s (mg.g^{-1})	136.737
		K_s (mg.L^{-1})	4.31E-5
		N_s	2.150
		R^2	0.985
		F_{error}	85.017
Khan	$q_e = \frac{q_s b_K C_e}{(1 + b_K C_e)^2 K}$	q_s	203.402
		a_k	1.123
		b_k	0.004
		R^2	0.962
		F_{error}	57.231
Toth	$q_e = \frac{K_T C_e}{(a_T + C_e)^{1/t}}$	K_t	395.127
		A_t	441.696
		R^2	0.962
		F_{error}	119.328
Redlich Peterson	$q_e = \frac{K_e C_e}{1 + a_R C_e^g}$	K_R (g.L^{-1})	0.895
		a_R (mg.L^{-1})	0.002
		g ($0 \leq g \leq 1$)	1.122
		R^2	0.962
		F_{error}	115.986

Table S8. Models, equations, and estimated parameters for isotherm models - dissulfoton.

Isotherm model	Equation	Parameter	Data
Langmuir	$q_e = \frac{q_s K_l C_e}{1+bC_e}$	q_s (mg g^{-1})	556.967
		K_L (mg L^{-1})	3.50E-4
		R^2	0.867
		F_{error}	53.517
Freundlich	$q_e = K_f C_e^{1/ns}$	K_F (mg.g^{-1}) (mg.L^{-1})^n	1,798
		n_F	1,620
		R^2	0.791
		F_{error}	168.515
Sips	$q_e = \frac{q_s K_s C_e^{1/ns}}{1 + a_s C_e^{1/ns}}$	q_s (mg.g^{-1})	3.935
		K_s (mg.L^{-1})	6.71E-13
		N_s	360.895
		R^2	0.997
		F_{error}	95.052
Khan	$q_e = \frac{q_s b_K C_e}{(1 + b_K C_e)^2 K}$	q_s	856.769
		a_k	1.48E1
		b_k	1.67E-4
		R^2	0.935
		F_{error}	63.986
Toth	$q_e = \frac{K_T C_e}{(a_T + C_e)^{1/t}}$	K_t	4.29E19
		A_t	2.99E20
		R^2	0.935
		F_{error}	133.412
Redlich Peterson	$q_e = \frac{K_e C_e}{1 + a_R C_e^g}$	K_R (g.L^{-1})	0.143
		a_R (mg.L^{-1})	3.55E-21
		g ($0 \leq g \leq 1$)	5.443
		R^2	0.935
		F_{error}	129.676

Table S9. Models, equations, and estimated parameters for isotherm models - chlorpyrifos.

Isotherm model	Equation	Parameter	Data
Langmuir	$q_e = \frac{q_s K_l C_e}{1+bC_e}$	q_s (mg g^{-1})	9.980
		K_L (mg L^{-1})	0.004
		R^2	0.947
		F_{error}	78.175
Freundlich	$q_e = K_f C_e^{1/ns}$	K_F (mg.g^{-1}) (mg.L^{-1})^n	0.974
		n_F	3.711
		R^2	0.728
		F_{error}	257.764
Sips	$q_e = \frac{q_s K_s C_e^{1/ns}}{1 + a_s C_e^{1/ns}}$	q_s (mg.g^{-1})	1.693
		K_s (mg.L^{-1})	9.69E-5
		N_s	9.196
		R^2	0.972
		F_{error}	77.610
Khan	$q_e = \frac{q_s b_K C_e}{(1 + b_K C_e)^2 K}$	q_s	19.421
		a_k	1.396
		b_k	0.001
		R^2	0.997
		F_{error}	52.245
Toth	$q_e = \frac{K_T C_e}{(a_T + C_e)^{1/t}}$	K_t	22.157
		A_t	692.752
		R^2	0.988
		F_{error}	108.931
Redlich Peterson	$q_e = \frac{K_e C_e}{1 + a_R C_e^g}$	K_R (g.L^{-1})	0.020
		a_R (mg.L^{-1})	6.75E-5
		g ($0 \leq g \leq 1$)	1.395
		R^2	0.997
		F_{error}	105.880

Optimization of the extraction conditions

Table S10 presents the factorial design matrix with both real and coded values for the multiple response analysis.

Table S10. Fractional factorial design.

Experiments	Conditining pH	Sample pH	Mass (mg)	Sample volume	Eluent volume	RM
1	3 (-1)	3 (-1)	25 (-1)	1 (-1)	2 (1)	0.144
2	11 (1)	3 (-1)	25 (-1)	1 (-1)	0,5 (-1)	0.411
3	3 (-1)	11 (1)	25 (-1)	1 (-1)	0,5 (-1)	0.467
4	11 (1)	11 (1)	25 (-1)	1 (-1)	2 (1)	0.111
5	3 (-1)	3 (-1)	200 (1)	1 (-1)	0,5 (-1)	0.653
6	11 (1)	3 (-1)	200 (1)	1 (-1)	2 (1)	0.180
7	3 (-1)	11 (1)	200 (1)	1 (-1)	2 (1)	0.125
8	11 (1)	11 (1)	200 (1)	1 (-1)	0,5 (-1)	0.391
9	3 (-1)	3 (-1)	25 (-1)	10 (1)	0,5 (-1)	2.750
10	11 (1)	3 (-1)	25 (-1)	10 (1)	2 (1)	0.820
11	3 (-1)	11 (1)	25 (-1)	10 (1)	2 (1)	0.345
12	11 (1)	11 (1)	25 (-1)	10 (1)	0,5 (-1)	2.088
13	3 (-1)	3 (-1)	200 (1)	10 (1)	2 (1)	1.464
14	11 (1)	3 (-1)	200 (1)	10 (1)	0,5 (-1)	2.626
15	3 (-1)	11 (1)	200 (1)	10 (1)	0,5 (-1)	1.863
16	11 (1)	11 (1)	200 (1)	10 (1)	2 (1)	0.791
17	7 (0)	7 (0)	100 (0)	5 (0)	1 (0)	0.991
18	7 (0)	7 (0)	100 (0)	5 (0)	1 (0)	1.129
19	7 (0)	7 (0)	100 (0)	5 (0)	1 (0)	1.102

Figure S3 shows the Pareto chart obtained from a 2^{5-1} fractional factorial design with five variables: eluent volume, sample pH, conditioning pH, sample volume, and sorbent mass.

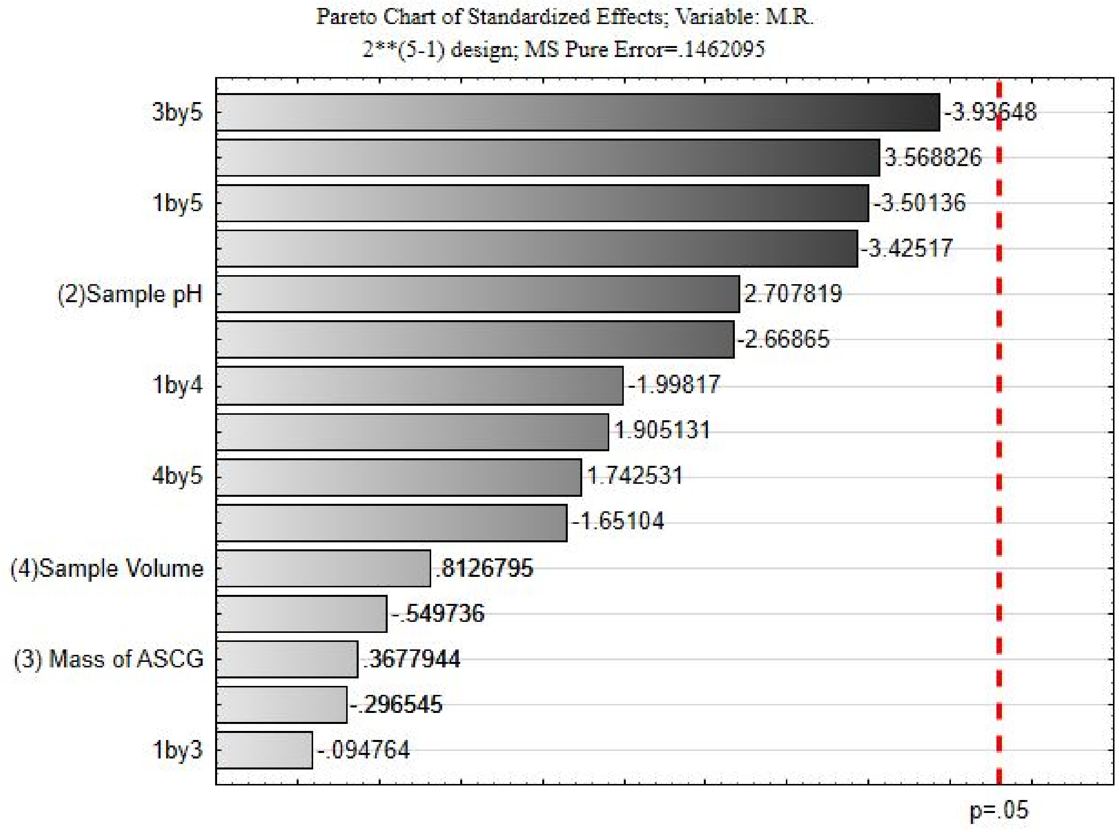

Figure S3. Pareto chart obtained from the 2^{5-1} fractional factorial design.
